# Gypenosides ameliorate ductular reaction and liver fibrosis *via* inhibition of hedgehog signaling

**DOI:** 10.3389/fphar.2022.1033103

**Published:** 2022-11-22

**Authors:** Yonghong Hu, Xiaoli He, Xiaoxi Zhou, Yue Liang, Yadong Fu, Linzhang Zhang, Jing Fang, Wei Liu, Gaofeng Chen, Yongping Mu, Hua Zhang, Hong Cai, Chenghai Liu, Ping Liu, Jiamei Chen

**Affiliations:** ^1^ Institute of Liver Diseases, Key Laboratory of Liver and Kidney Diseases (Ministry of Education), Shuguang Hospital Affiliated to Shanghai University of Traditional Chinese Medicine, Shanghai, China; ^2^ Shanghai Key Laboratory of Traditional Chinese Clinical Medicine, Shanghai, China; ^3^ Institute of Interdisciplinary Integrative Medicine Research, Shanghai University of Traditional Chinese Medicine, Shanghai, China; ^4^ Xiamen Hospital of Traditional Chinese Medicine, Xiamen, Fujian, China

**Keywords:** gypenosides, liver fibrosis, ductular reaction, hedgehog signaling, NPLC0393

## Abstract

**Backgroud and aims:** Ductular reaction (DR) is a common pathological change and thought to have a key role in the pathogenesis and progression of liver fibrosis. Our previous study reported Gypenosides (GPs) ameliorated liver fibrosis, however, the anti-fibrotic mechanisms of GPs are still unclear.

**Methods:** Liver fibrosis was induced in rats by carbon tetrachloride combining with 2-acerylaminofluorene (CCl_4_/2-AAF), and Mdr2 knockout (*Mdr2* ^−/−^) mice to evaluate the anti-fibrotic role of GPs. *In vitro*, WB-F344 cells, a hepatic progenitor cells (HPCs) line, with or without Gli1 overexpressing lentiviral vectors, were induced by sodium butyrate (SB) to validate the mechanism of GPs and NPLC0393, the main ingredient of GPs.

**Results:** Both in CCl_4_/2-AAF-treated rats and *Mdr2* ^−/−^ mice, GPs obviously reduced the deposition of collagen and hydroxyproline content, inhibited the activation of hepatic stellate cells and inflammatory cell infiltration. Notably, GPs reduced the expressions of Epcam, CK19, CK7, Dhh, Smo, Ptch2, Gli1 and Gli2. Furthermore, CK19^+^ cells co-expressed Gli1, while the number of CK19^+^/Gli1^+^ cells was decreased by GPs. *In vitro*, GPs and NPLC0393 inhibited the differentiation of WB-F344 cells toward a biliary phenotype. Mechanistically, GPs and NPLC0393 protected against DR by inhibiting hedgehog signaling, which was supported by the results that DR, triggered directly by Gli1 overexpressing lentiviral vector was blocked by administration with GPs or NPLC0393.

**Conclusion:** GPs attenuated DR and liver fibrosis by inhibiting hedgehog signaling, which provided more evidences and a novel mechanism of anti-fibrotic effect of GPs.

## 1 Introduction

Liver fibrosis, which is characterized by excessive deposition of extracellular matrix and the formation of fibrous scars (Parola and Pinzani, 2019), is a common pathological consequence of chronic liver injuries induced by various etiological factors, such as virus infection, alcoholism, chemical damage, and autoimmune disorders (Parola and Pinzani, 2019; Roehlen et al., 2020). If untreated, it ultimately leads to cirrhosis, end-stage liver diseases, and even hepatocellular carcinoma (HCC), becoming a major health challenge worldwide (Zhao et al., 2018; Asrani et al., 2019). Ductular reaction (DR) has been observed in acute and chronic liver injuries. It has been reported that in cholestatic liver injury, chronic viral hepatitis, and alcoholic hepatitis, ductular reactive cells extended into the hepatic parenchyma and played a key role in recruiting immune cells to create a proinflammatory and profibrogenic microenvironment, which correlated with the severity of fibrosis and short-term survival (Clouston et al., 2005; Zhao et al., 2018; Aguilar-Bravo et al., 2019). In our previous study, we demonstrated that abnormal proliferated cholangioctyes formed DR, exhibiting a profibrotic effect in rats with bile duct ligation (BDL), and the extent of DR was increased with the prolongation of molding time (Zhang et al., 2016). Although experimental data has proved that liver fibrosis, even early cirrhosis, is reversible through pharmacologic treatment or gene-editing technology. However, there are currently no effective FDA-approved drugs targeting liver fibrosis. Therefore, investigations searching for effective herbal medicines or bioactive compounds for anti-hepatic fibrosis have been a longstanding goal.

The Hedgehog (Hh) signaling is known to play a critical role in various acute and chronic liver diseases, including 70% partial hepatectomy (Ochoa et al., 2010), HBV/HCV infection (Pereira Tde et al., 2010), cholestatic liver injury (Gupta et al., 2020), alcoholic liver disease (Jung et al., 2008) and non-alcoholic fatty liver disease (Syn et al., 2012). Although temporary Hh signaling activation is required for efficient liver regeneration, dysregulated activation of Hh signaling in liver injuries encourages the development of the liver fibrosis and cirrhosis. As a result, inhibiting Hh signaling offers hope for the therapy of liver fibrosis.

Gypenosides (GPs) is one of the main pharmacologically active components extracted from *Gynostemma pentaphyllum* (Cui et al., 1999). GPs had effects on regulating lipid metabolism (Zhang et al., 2021), anti-inflammatory (Tu et al., 2021), anti-oxidative (Zhang et al., 2018), anti-cancer (Li et al., 2022), anti-diabetes (He et al., 2015), and hepatoprotection (Li et al., 2020). GPs have significant therapeutic effects on a variety of animal models of liver fibrosis. In addition, prior work has shown that the anti-fibrotic mechanisms of GPs were related to inhibiting the proliferation and activation of hepatic stellate cells (HSCs) (Chen et al., 2008), as well as reducing the damage caused by aldehydes and lipid peroxidation (Song et al., 2017). However, little work has been focused on the effect of GPs on DR.

In the present study, two rodent models of hepatic fibrosis induced by different pathogenic processes, including rats treated with carbon tetrachloride (CCl_4_) combined with 2-acetylaminofluorene (2-AAF) and multidrug resistance gene 2 knockout (*Mdr2* ^−/−^) mice, and an *in vitro* model of differentiation of hepatic progenitor cells (HPCs) into cholangiocytes by sodium butyrate (SB) exposure, were used to elucidate the effect and mechanism of GPs on liver fibrosis. The findings demonstrated that GPs ameliorated DR and liver fibrosis by inhibiting hedgehog signaling.

## 2 Materials and methods

### 2.1 Animals

Female Fisher 344 rats, 6–8 weeks old, with 160–180 g, were purchased from Beijing Vital River Laboratory Animal Technology (Beijing, China). *Mdr2* ^−/−^ and wild-type (WT) mice were purchased from the Shanghai Research Center of Southern Model Organisms (Shanghai, China). All animals were housed at the Animal Experiment Center of Shanghai University of Traditional Chinese Medicine, with a 12-h light-dark cycle and free access to a chow diet and water. All animal experiments were approved by the Experimental Animal Ethics Committee of Shanghai University of Traditional Chinese Medicine (NO. PZSHUTCM190505002, and NO. PZSHUTCM200731007).

### 2.2 Drugs

CCl_4_ (10006464) was purchased from Sinopharm Chemical Reagent Co. Ltd. (Shanghai, China). 2-AAF (53-96-3), GANT61 (G9048), and SB (B5887) were purchased from Sigma-Aldrich Co., Ltd. (America) GPs (180527) was purchased from Shanghai Winherb Medical Technology Co., Ltd. (Shanghai, China). NLPC0393 was gifted by Dr. Shanhua Fang from Shanghai Institute of Materia Medica, Chinese Academy of Sciences.

### 2.3 Liver fibrosis animal models and treatment with GPs

#### 2.3.1 CCl_4_ combined with 2-AAF -treated rats

All Fisher 344 rats were randomly divided into the CCl_4_-injected group and the normal control group. The CCl_4_-injected group was injected subcutaneously with 30% CCl_4_ -olive oil solution at a dose of 2 ml/kg, body weight, twice a week for 6 weeks. At the beginning of the seventh week, all CCl_4_-injected rats were randomly divided into three groups, including the CCl_4_ combined with 2-AAF (10 mg/kg/d) -treated group (the CCl_4_/2-AAF-treated group), the CCl_4_/2-AAF plus GPs (100 mg/kg/d) -treated group (the GPs-treated group), and the CCl_4_/2-AAF plus GANT61 (25 mg/kg/qod) -treated group (the GANT61-treated group). All drugs were administered orally according to different groups up to the end of the ninth week.

#### 2.3.2 *Mdr2* ^−/−^ spontaneous primary sclerosing cholangitis model

A deficiency of canalicular phospholipid translocase in *Mdr2* ^−/−^ mice, resulted in lacking phospholipids in the bile further leading to the accumulation of toxic bile acids in hepatocytes and initiation of a profibrogenic ductular reaction, and spontaneously progressing to severe biliary fibrosis, resembling PSC (Popov et al., 2005). From the beginning of the eighth week after birth, *Mdr2* ^−/−^ mice were randomly divided into the *Mdr2* ^−/−^ model group and the GPs (200 mg/kg/d) -treated group. All mice were sacrificed at the end of the 11th week, liver tissues were collected for further experiments.

### 2.4 WB-F344 cells culture and treatment

WB-F344 cells, the HPC line, was purchased from Xiangf Bio (Shanghai, China), and cultured in high glucose DMEM supplemented with 10% fetal bovine serum, at 37 °C humidified atmosphere with 5% CO_2_. Cells were stimulated with 3.75 μM SB for 4 days to induce the differentiation into cholangiocytes, meanwhile treated for 4 days with or without GPs and NPLC0393 dissolved in DMSO with appropriate concentrations.

### 2.5 *Gli1* overexpressing lentivirus vectors

The *Gli1* gene overexpressing lentivirus vector and empty control lentivirus vector were prepared by Shanghai Genechem Co. Ltd. (Shanghai, China). The lentivirus vector carried a green fluorescent label (Vector name, GV367; element sequence, Ubi-MCS-SV40-EGFP-IRES-puromycin; control serial number, CON238). WB-F344 cells at the concentration of 1 × 10^4 cell/mL were cultured in 12-well plates for 24 h and treated with lentivirus vector per well at a MOI = 50. After 12 h, the transfection medium was changed to the medium according to the experimental groups. Cells were collected for the subsequent examination after 72 h.

### 2.6 Histopathological and immunohistochemical analysis

Liver tissues were fixed in 10% neutral-buffered formalin solution for at least 48 h. Subsequently, liver tissues were paraffin-embedded and cut at a thickness of 4 μm for hematoxylin and eosin (H&E) and Sirius red (SR) staining according to the manufacturer’s instructions.

Immunohistochemical staining was performed with the standard methods. Liver sections were firstly deparaffinized and washed, retrieved the antigens in citrate buffer, and inactivated the endogenous peroxidases with 3% hydrogen peroxide. Then blocked by 10% goat serum or 5% bovine serum, and incubated with primary antibodies at 4°C overnight. After three washes in PBST, sections were incubated with the corresponding secondary antibodies conjugated with HRP (GTVision III Immunohistochemical Detection Kit, HRP/DAB, anti-mouse/rabbit IgG, two-step, GK5005/5007, Gene Tech, Shanghai, China) for 30 min at 37°C in next day. Sections were then washed, colored with DAB, counterstained with hematoxylin, washed, dehydrated, and sealed. The staining images were acquired by a Leica SCN 400 slide scanner (Leica Microsystems Ltd., Mannheim, Germany) or bright-field microscopy (Olympus, Beijing, China). The detailed antibodies were provided in Supplementary Table S1.

### 2.7 Immunofluorescence analysis

Liver sections (8 μm) were fixed in pre-cooled (−20°C) acetone and cells were fixed in 4% paraformaldehyde for 10 min. After fixed, tissue sections and cells were treated with blocking solution (10% goat serum or 5% bovine serum in PBS) for 30–60 min at room temperature. Subsequently, primary antibodies were incubated at 4°C overnight. After three washes in PBST (5 min per wash), secondary antibodies were incubated at 37°C for 30 min in the next day. And 4′,6-diamidino-2 phenylindole (DAPI) was labeled the nuclear visualization. Fluorescent images were captured by a DP80 fluorescence inverted microscope (Olympus, Beijing, China) or a laser-scanning confocal microscope (Olympus, Beijing, China). The detailed antibodies were provided in Supplementary Table S2.

### 2.8 Western blot analysis

The liver tissues and cells were lysed by lysis buffer with protease and phosphatase inhibitors. After centrifugation (12000 rpm, 15 min) at 4°C, the supernatant was collected and the protein concentration was determined using the BCA assay. The samples were then mixed with 5× loading buffer and heated at 100°C for 8 min, fractionated by SDS-PAGE, and transferred to polyvinylidene fluoride (PVDF) membranes. The membranes were then incubated in blocking solution (5% bovine serum in PBS) for 60 min at RT, followed by overnight incubation at 4°C with the primary antibodies. After three washes in PBST (5 min each), the membrane was incubated with the appropriate secondary antibodies at RT for 1 h. After three more washes (5 min per wash) in PBST, the membranes were imaged by the Odyssey infrared imaging system (LiCor, America) or the ECL system (Tanon, Shanghai, China). The detailed antibodies were provided in Supplementary Table S3.

### 2.9 Quantitative real-time quantitative PCR

Quantitative real-time PCR was performed according to the manufacturer’s instructions as follows. Total RNA was extracted from liver tissues and cells using the MagExtraction™ RNA kit (TOYOBO, NPK-201F). The total RNA was reverse transcribed into cDNA by ReverTra Ace qPCR RT Master Mix with gDNA Remover (TOYOBO, FSQ-301). Real-time quantitative PCR was performed using the SYBR^®^ Green Premix Pro Taq HS qPCR Kit (Accurate Biology, AG11701). The primer sequences used are listed in Supplementary Table S4 and Supplementary Table S5.

### 2.10 Statistical analysis

The data were subjected to statistical analysis including Student’s t-test when appropriate or univariate analysis of variance (ANOVA) when more than two groups were compared, using the IBM SPSS 26.0 statistical package. *p*-value < 0.05 was considered significant.

## 3 Results

### 3.1 GPs ameliorated CCl_4_/2-AAF-induced liver fibrosis in rats

**FIGURE 1:**
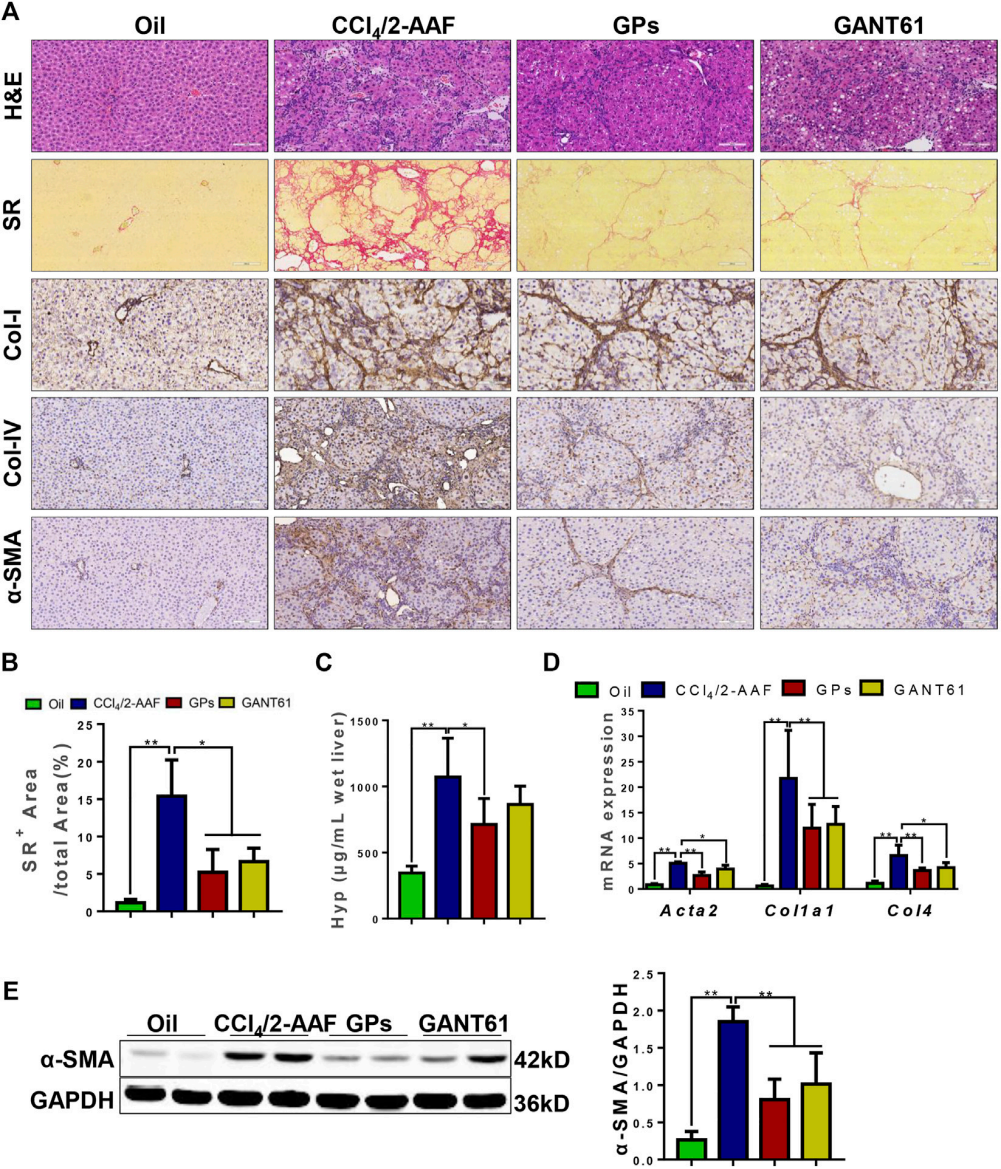
GPs alleviated CCl_4_/2-AAF-induced liver fibrosis in rats. **(A)** Representative images of liver sections stained with H&E (200×), SR (100×), and immunohistochemical staining (200×). **(B)** Morphometric quantification of the SR-positive area (%). **(C)** The Hyp content. **(D)** The mRNA expressions of *Atca2*, *Col1a1,* and *Col4*. **(E)** Western blotting and the gray-level score for α-SMA. *, *p* < 0.05; **, *p* < 0.01. Oil: the control group; CCl_4_/2-AAF: the CCl_4_ combined with 2-AAF-treated group; GPs: the GPs-treated group; GANT61: the GANT61-treated group.

To demonstrate whether GPs can improve liver fibrosis, a rat liver fibrosis model induced by CCl_4_/2-AAF was first established. In this model, 2-AAF could inhibit hepatocyte proliferation, conversely promote the activation and proliferation of HPCs. H&E staining showed apparent damaged lobules, extensive inflammatory cell infiltration in the portal area, and fibrous septa were visible in the CCl_4_/2-AAF-treated group. However, necroinflammatory hepatic lesions were markedly attenuated in the GPs or GANT61-treated group compared to the CCl_4_/2-AAF-treated group (Figure 1A). SR staining revealed that a large amount of collagen fiber deposited and some extended into the liver parenchyma, which formed pseudo nodules in the CCl_4_/2-AAF-treated group, in contrast, collagen deposition was significantly decreased after GPs or GANT61 treatment (Figure 1A). Consistently, the ratio of collagen-positive area and the hepatic Hyp content in the CCl_4_/2-AAF-treated group were significantly increased (*p* < 0.01), which were remarkably decreased in the GPs or GANT61-treated group (*p* < 0.05) (Figures 1B,C **)**. Immunostaining showed that in the CCl_4_/2-AAF-treated group, collagen type I (Col-I), Col-IV, and alpha-smooth muscle actin (α-SMA) were mainly expressed in the fibrotic septa, and increased compared to the normal group, while GPs and GANT61 significantly reduced the expression of Col-I, Col-IV and α-SMA (Figure 1A). Moreover, *Col1a1*, *Col4,* and *Acta2* mRNA expressions were significantly increased in the CCl_4_/2-AAF-treated group compared to the normal group (*p* < 0.01), whereas their expressions were decreased after administration with GPs or GANT61 (*p* < 0.01, *p* < 0.05) (Figure 1D). In addition, the α-SMA protein expression was significantly lower in the GPs-treated group and the GANT61-treated group than that in the CCl_4_/2-AAF-treated group (*p* < 0.01) (Figure 1E). These results demonstrated that GPs and GANT61 significantly alleviated liver fibrosis induced by CCl_4_/2-AAF in rats.

### 3.2 GPs ameliorated liver inflammation and collagen deposition in *Mdr2* ^*−/−*^ mice

**FIGURE 2:**
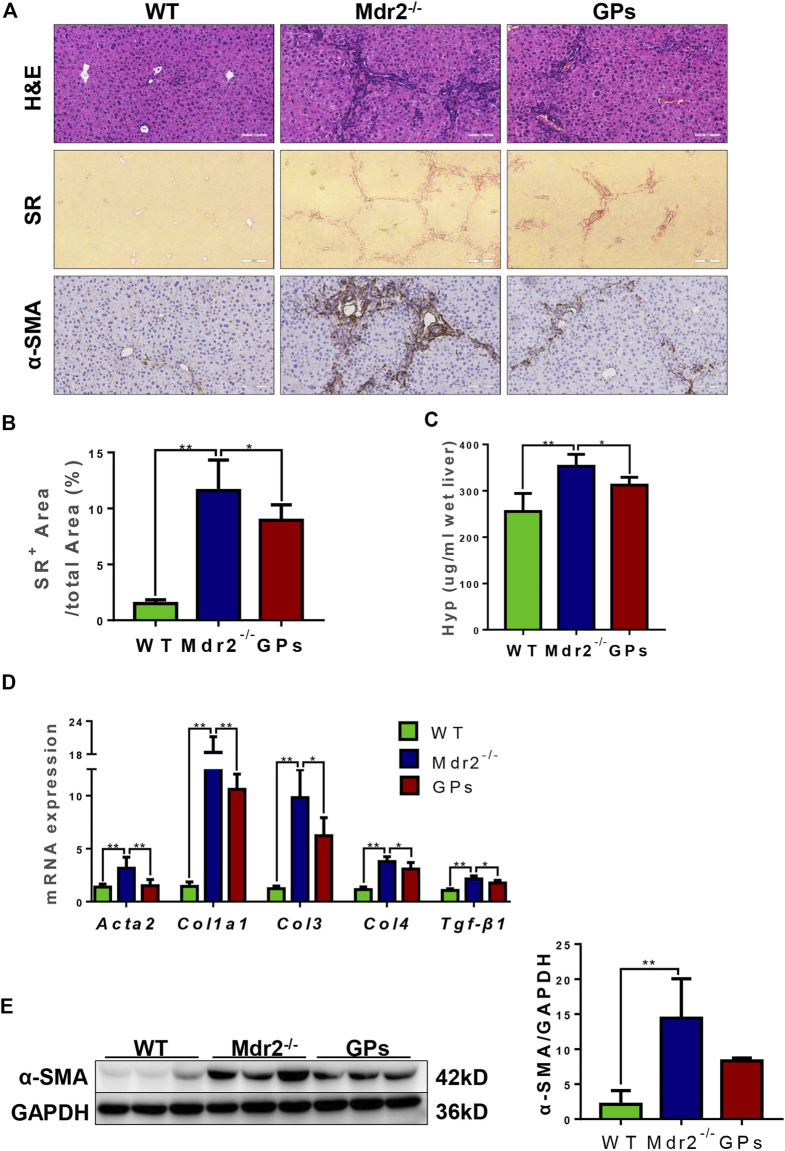
GPs ameliorated liver inflammation and collagen deposition in *Mdr2* ^*−/−*^ mice. **(A)** Representative images of liver sections stained with H&E (200×) and SR (100×), and immunohistochemical staining for α-SMA (200×). **(B)** Morphometric quantification of the SR-positive area (%). **(C)** The Hyp content. **(D)** The relative mRNA expressions of *Atca2*, *Col1a1*, *Col3*, *Col4,* and *Tgf-β1*. **(E)** Western blotting and the gray-level score for CK19. 0.05; **, *p* < 0.01. Mdr2^+/−^: the WT control group; Mdr2^−/−^: the Mdr2^−/−^ mice group; GPs: the GPs-treated group.

The anti-fibrotic effect of GPs was continued to investigate in another hepatic fibrosis model, *Mdr2* ^*−/−*^ mice, which could spontaneously develop cholestatic liver injury and fibrosis. Similar to the results found in the CCl_4_/2-AAF-treated rats, H&E staining also showed the hepatic lobule structure was disorganized and inflammatory cells infiltrated in *Mdr2* ^*−/−*^ mice, which were markedly improved after administration with GPs (Figure 2A). SR staining revealed that collagen deposition was increased, forming bridging fibrosis and complete pseudo nodule in *Mdr2* ^*−/−*^ mice, which was attenuated in GPs-treated group (Figure 2A). In addition, the Hyp content and SR-positive area were extensively reduced in GPs-treated group compared with the *Mdr2* ^*−/−*^ group (*p* < 0.05) (Figures 2B,C). Immunostaining showed that α-SMA expression in the liver was increased in *Mdr2* ^*−/−*^ mice, while significantly reduced after GPs administration (*p* < 0.01) (Figure 2E), which was consistent with mRNA expression of *Acta2* analyzed by qRT-PCR (Figure 2D). Besides that, GPs inhibited the mRNA expression of *Col1a1*, *Col3*, *Col4* and *Tgf-β1* (*p* < 0.01, *p* < 0.05) (Figure 2D). All these results proved that GPs alleviated liver fibrosis in *Mdr2* ^*−/−*^ mice.

### 3.3 GPs suppressed ductular reaction in fibrotic animals

**FIGURE 3:**
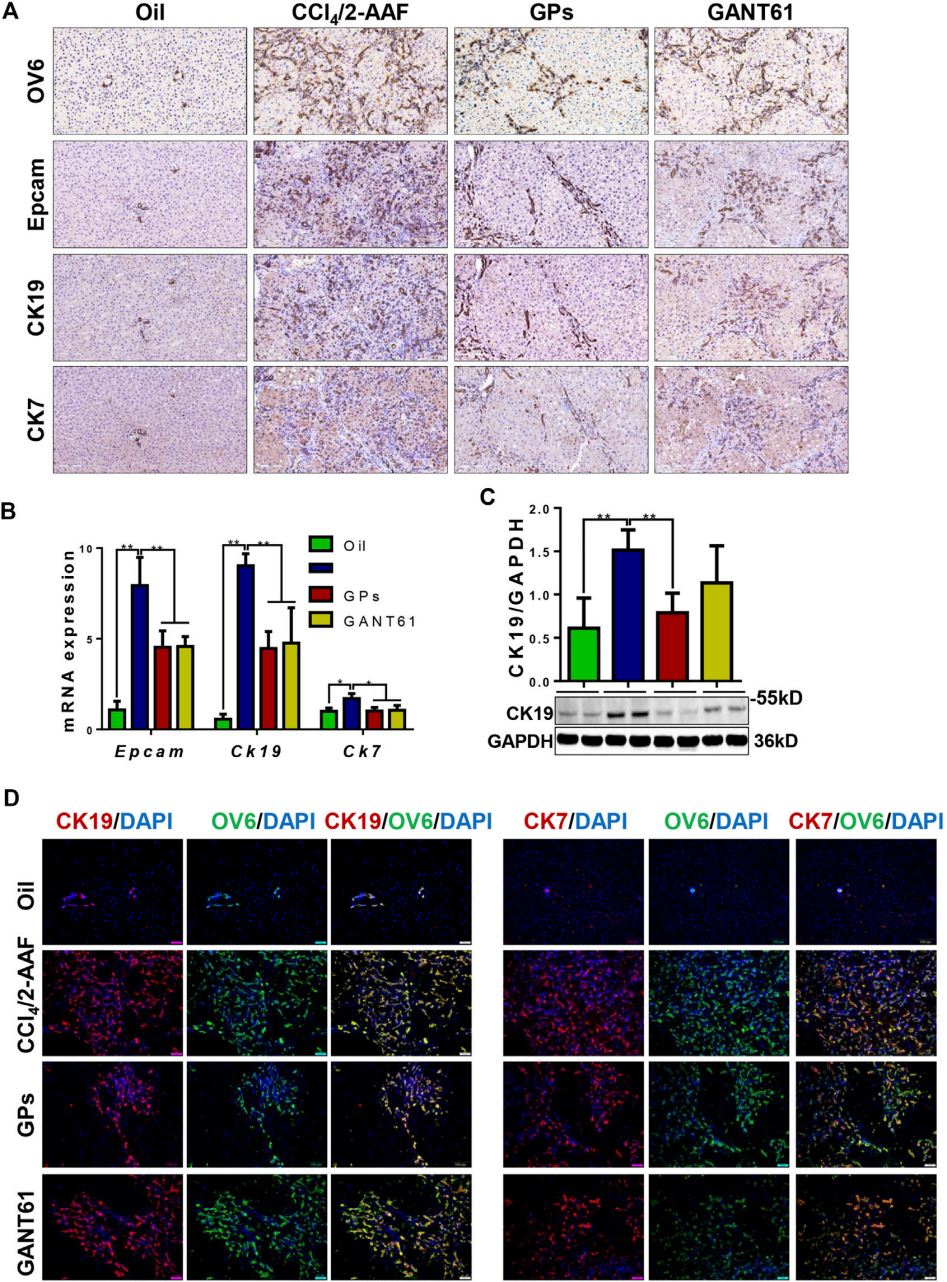
GPs suppressed DR in CCl_4_/2-AAF-induced rats. **(A)** Representative immunohistochemical staining for OV6, Epcam, CK19, and CK7 (200×). **(B)** The mRNA expressions of *Epcam*, *Ck19*, and *Ck7.* **(C)** Western blotting and the gray-level score for CK19. **(D)** Confocal analysis of co-staining for CK19 (red) and OV6 (green), CK7 (red) and OV6 (green) (200×). *, *p* < 0.05; **, *p* < 0.01. Oil: the control group; CCl_4_/2-AAF: the CCl_4_ combined with 2-AAF-treated group; GPs: the GPs-treated group; GANT61: the GANT61-treated group.

**FIGURE 4:**
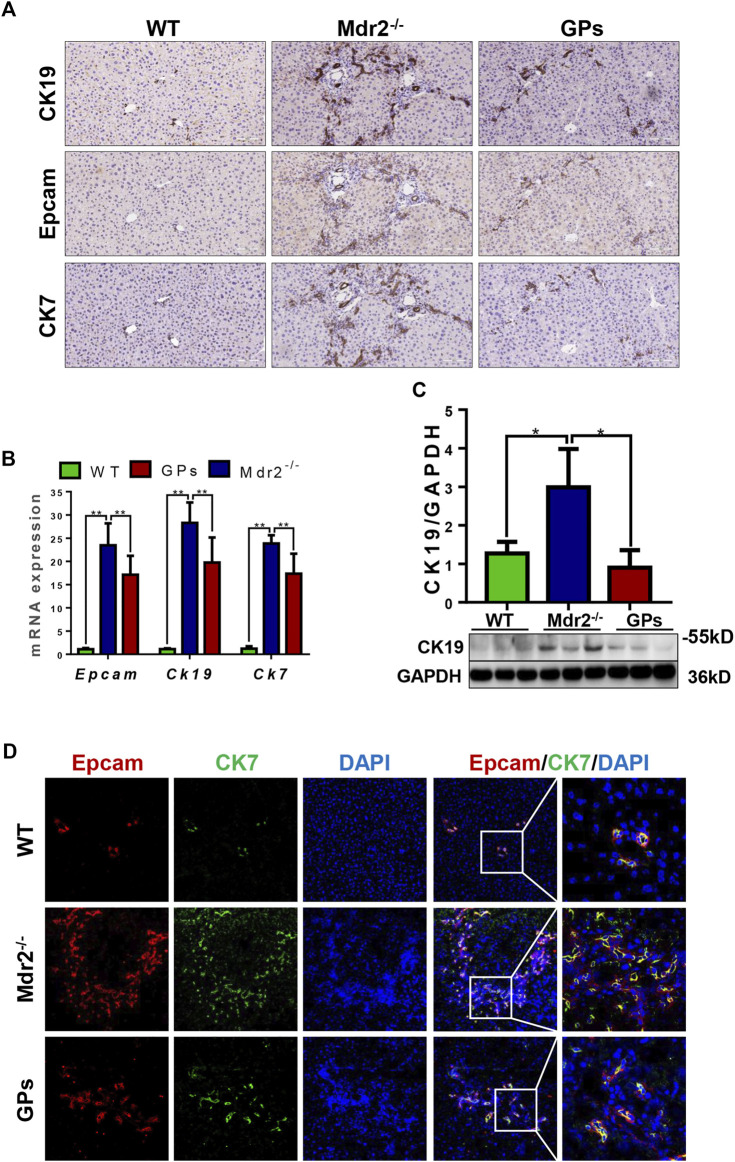
GPs inhibited DR in CCl_4_/2-AAF-induced rats. **(A)** Representative immunohistochemical staining for CK19, Epcam, and CK7 (200×). **(B)** The mRNA expressions of *Epcam, Ck19*, and *Ck7*. **(C)** Western blotting and gray-level score of CK19. **(D)** Confocal analysis of co-staining for Epcam (red) and CK7 (green) (200×). *, *p* < 0.05; **, *p* < 0.01. Mdr2^+/−^: the WT control group; Mdr2^−/−^: the Mdr2^−/−^ mice group; GPs: the GPs-treated group.

DR is associated with liver fibrosis and the extent of DR parallels patient mortality (Sato et al., 2019). Immunostaining showed that OV6, an antigen-specific for rodent HPCs, was mainly expressed in the portal area in the normal group. In the CCl_4_/2-AAF-treated rats, OV6 was widely found in the injured liver, which was lower in the GPs or GANT61-treated group. For analyzing the changes in DR, serial sections were stained with epithelial cell adhesion molecule (Epcam), keratin19 (CK19), and CK7 by immunohistochemistry, which were biomarkers of HPCs and cholangiocytes, respectively. In the CCl_4_/2-AAF-treated group, Epcam was expressed in HPCs and reactive cholangiocytes located in the fibrous septa and occasionally in the scattered cells within hepatic lobules. CK19 and CK7 were expressed in pre-existing and reactive cholangiocytes. Further observation revealed that Epcam^+^ cells also expressed CK19 and CK7. While the expression of Epcam, CK19, and CK7 were decreased in the GPs-treated and GANT61-treated groups (Figure 3A). The mRNA expression of *Epcam*, *Ck19*, and *Ck7*, as well as the protein expression of CK19, were increased in the CCl_4_/2-AAF-treated group, which were significantly reduced after administration of GPs or GANT61 (*p* < 0.01, *p* < 0.05) (Figures 3B,C). Moreover, double-immunofluorescence staining showed that CK19, CK7, and OV6 were co-localized in reactive cholangiocytes, while the numbers of CK19^+^/OV6^+^ and CK7^+^/OV6^+^ cells were reduced in the GPs-treated and GANT61-treated groups compared with the CCl_4_/2-AAF-treated group (Figure 3D). Consistently with our finding in CCl_4_/2-AAF-treated rats, GPs also reduced the expressions of CK19, Epcam, and CK7 in *Mdr2* ^*−/−*^ mice (*p* < 0.01, *p* < 0.05) (Figures 4A–C). And the number of Epcam^+^/CK7^+^ cells was obviously decreased after GPs treatment analyzed by double-immunofluorescence staining (Figure 4D). These results demonstrated that GPs inhibited DR in fibrotic models.

### 3.4 GPs suppressed the activation of hedgehog signaling *in vivo*

**FIGURE 5:**
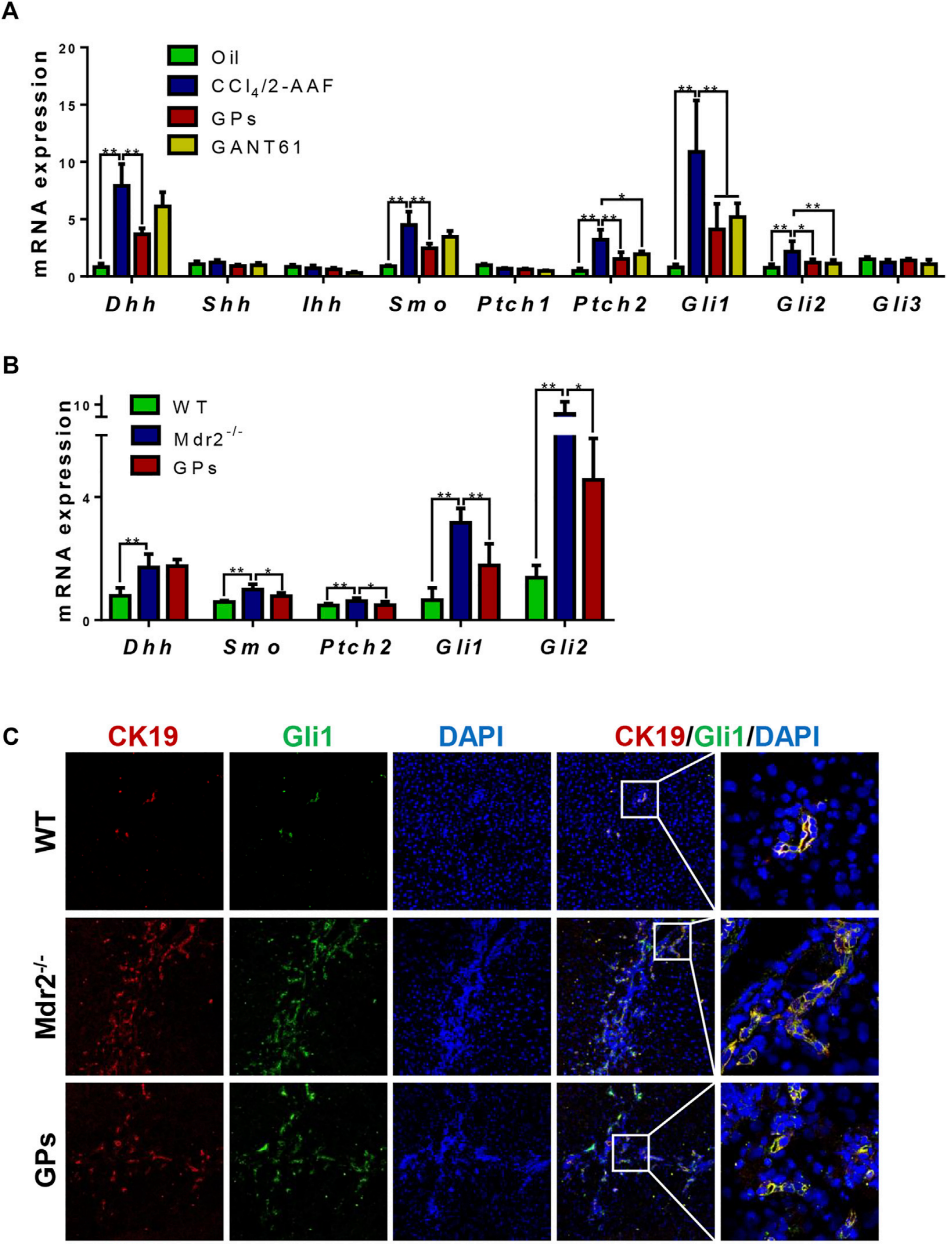
GPs attenuated DR via hedgehog pathway in fibrotic animals. **(A)** The mRNA expressions of *Dhh*, *Shh*, *Ihh*, *Smo*, *Ptch1*, *Ptch2*, *Gli1*, *Gli2*, and *Gli3* in CCl_4_/2-AAF-induced rats. **(B)** The mRNA expressions of *Dhh*, *Smo*, *Ptch2*, *Gli1*, and *Gli2* in *Mdr2* ^*−/−*^ mice. **(C)** Confocal analysis of co-staining for CK19 (red) and Gli1 (green) (200×). *, *p* < 0.05; **, *p* < 0.01.

In CCl_4_/2-AAF-treated rats, qRT-PCR showed that the mRNA expressions of *Dhh*, *Smo*, *Ptch2*, *Gli1,* and *Gli2*, the relative genes of hedgehog signaling, were upregulated in the CCl_4_/2-AAF-treated rats (*p* < 0.01), which were downregulated in the GPs-treated group (*p* < 0.01, *p* < 0.05) (Figure 5A). GANT61, a direct small-molecule inhibitor of Gli1/Gli2, obviously reduced the mRNA expression of *Gli1, Gli2* and *Ptch2* (*p* < 0.01, *p* < 0.05) (Figure 5A). In addition, in *Mdr2* ^*−/−*^ mice, GPs decreased the expression of *Smo*, *Ptch2*, *Gli1* and *Gli2* (*p* < 0.01, *p* < 0.05) (Figure 5B). Importantly, double-immunofluorescence staining results showed Gli1 was expressed in the CK19^+^ reactive cholangiocytes, and the number of Gli1^+^/CK19^+^ cells was significantly increased in *Mdr2* ^*−/−*^ mice, which was obviously reduced in the GPs-treated group (Figure 5C). These results indicated that GPs played an anti-fibrotic role by inhibiting hedgehog signaling.

### 3.5 GPs inhibited the differentiation of HPCs into cholangiocytes by inhibition of hedgehog signaling *in vitro*

**FIGURE 6:**
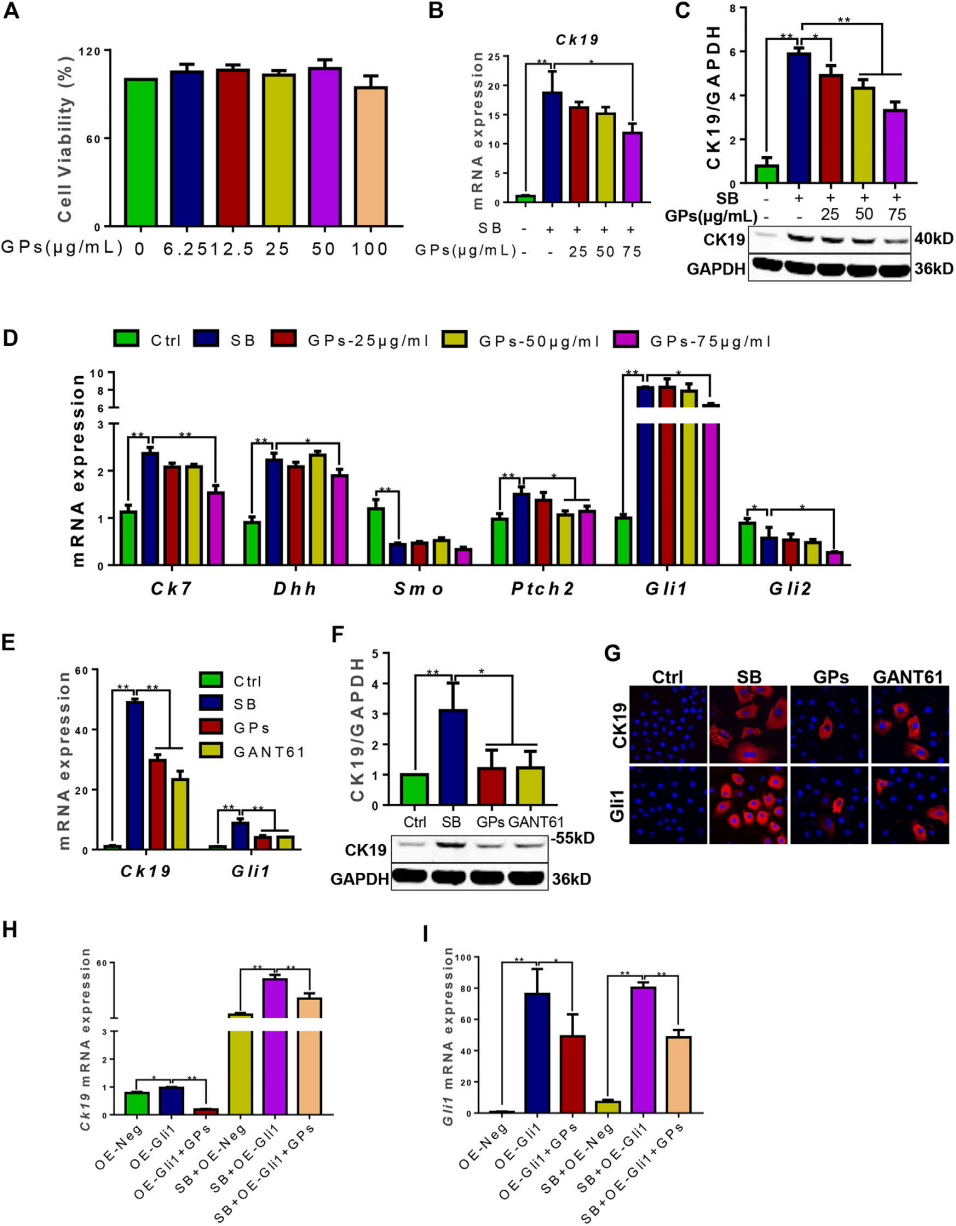
GPs inhibited the differentiation of WB-F344 cells into cholangiocytes via hedgehog signaling. **(A)** Cell viability of GPs on WB-F344 cells. **(B)** The mRNA expression of *Ck19*. **(C)** Western blotting and the gray-level score for CK19. **(D)** The mRNA expressions of *Ck7*, *Dhh, Smo*, *Ptch2*, *Gli1*, and *Gli2*. **(E)** The mRNA expressions of *Ck19* and *Gli1*. **(F)** Western blotting and the gray-level score for CK19. **(G)** Representative fluorescent cell images of WB-F344 cells stained with Gli1 and CK19 (600×). **(H)** The mRNA expression of *Ck19*. **(I)** The mRNA expression of *Gli1*. *, *p* < 0.05; **, *p* < 0.01.

To confirm whether GPs suppressed DR through hedgehog signaling, the *in vitro* model of differentiation of WB-F344 cells into cholangiocytes induced by SB was established. And the qRT-PCR result showed that the mRNA levels of *Dhh*, *Ptch2*, and *Gli1* were upregulated in the SB-treated group compared with the control group (*p* < 0.01) (Figure 6D), which suggested that Hh signaling plays an effect on the differentiation of WB-F344 cells into cholangiocytes. Based on the CCK8 assay showed that the different concentrations of GPs did not have a toxic effect on WB-F344 cells (Figure 6A). Then, GPs at the three concentrations (25, 50, 75 μg/ml) were selected to incubate WB-F344 cells for 4 days, and the results showed that GPs significantly decreased the expressions of CK19, CK7, Dhh, Ptch2 and Gli1 at 75 μg/ml compared with the SB-treated group (*p* < 0.01, *p* < 0.05) (Figures 6B–D), Further study found that the ability of GPs to inhibit CK19 and Gli1 expression was comparable to that of the Gli1 inhibitor, GANT61 (Figures 6E–G).

To further validate whether GPs inhibit the differentiation of WB-F344 cells into cholangiocytes in a Gli1-dependent manner, Gli1-overexpressing lentiviral vectors were transfected into WB-F344 cells. The results revealed that *Ck19* and *Gli1* expressions in the Gli1-overexpressing lentiviral vector-transfected group were upregulated compared with the empty vector-transfected group (*p* < 0.05, *p* < 0.01), which were remarkably downregulated after treatment with GPs (*p* < 0.05, *p* < 0.01) (Figures 6H,I). Even compared with the SB plus empty vector-transfected groups, the *Ck19* expression was notably increased in the SB plus Gli1-overexpressing lentiviral vector-transfected group (*p* < 0.01), which was also decreased after GPs treatment (*p* < 0.01) (Figure 6H). All these results demonstrated that Gli1 triggered and promoted the differentiation of WB-F344 cells into cholangiocytes, which could be inhibited by GPs.

### 3.6 NPLC0393, the natural compound of GPs, suppressed the differentiation of WB-F344 cells into cholangiocytes in a Gli1-dependent manner

**FIGURE 7:**
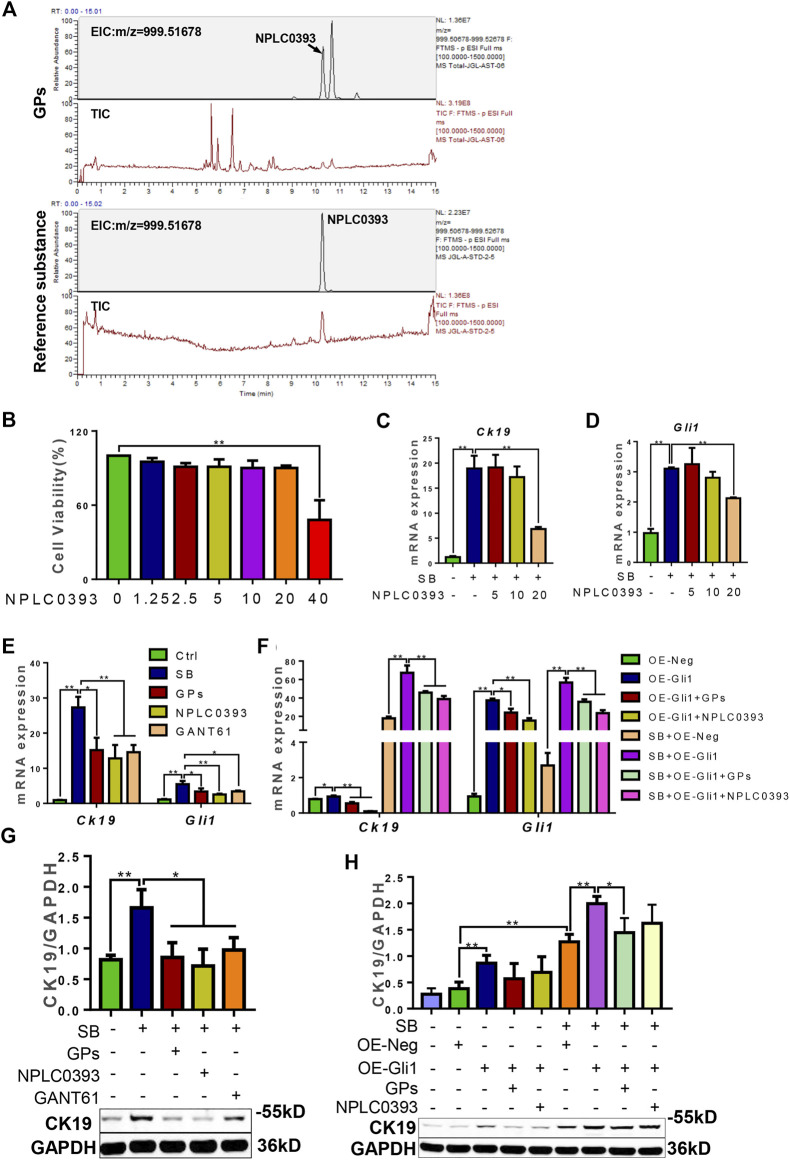
NPLC0393 suppressed the differentiation of WB-F344 cells toward a biliary phenotype in a Gli1-dependent manner. **(A)** The total ion chromatograms and extract ion chromatograms of NLPC0393 in GPs and reference substances (TIC and EIC). **(B)** Cell viability of NPLC0393 on WB-F344 cells. **(C)** The mRNA expressions of *Ck19*. **(D)** The mRNA expression of *Gli1*. **(E,F)** The mRNA expressions of *Ck19* and *Gli1*. **(G,H)** Western blotting and gray-level score of CK19. *, *p* < 0.05; **, *p* < 0.01.

NPLC0393 is a triterpene saponin constituent of GPs, which is isolated from the Chinese herb *Gynostemma pentaphyllum*. The total ion chromatograms (TIC) and extract ion chromatograms (EIC) of NLPC0393 in GPs and reference substances were shown in Figure 7A. The content of NPLC0393 in GPs was 2.6%. *In vitro* results showed that NPLC0393 at 20 μM reduced the expression of CK19 and Gli1 induced not only by SB stimulation but also by SB stimulation plus Gli1-overexpressing lentiviral vectors (*p* < 0.05, *p* < 0.01) (Figures 7B–H). In summary, NPLC0393, the compound of GPs also inhibited the differentiation of WB-F344 cells into cholangiocytes in a Gli1-dependent manner.

## 4 Discussion

Numerous candidates from Chinese herbs have been studied recently for treating liver fibrosis (Li, 2020). GPs are one of the triterpenoids mainly extracted from *Gynostemma pentaphyllum*. It has been reported that GPs had unambiguous hepatoprotective effects on liver injuries (Huang et al., 2019; Shen et al., 2020). Additionally, GPs significantly decreased the serum ALT and AST levels, ameliorated the histopathological changes in CCl_4_ or DMN-induced liver fibrosis models (Feng et al., 2012; Chen et al., 2017). In this study, the obvious anti-fibrotic effect of GPs was also demonstrated by other two different rodent models, the CCl_4_/2-AAF-induced hepatic fibrosis and *Mdr2* ^*−/−*^ mice. 2-AAF can block hepatocyte proliferation and induce the robust expansion of HPCs (Petersen et al., 1998). And the CCl_4_/2-AAF-induced DR-fibrosis model displays more severe fibrosis and even cirrhosis compared to the only CCl_4_-induced fibrotic model, which recapitulated the events commonly observed in human fibrosis (Chobert et al., 2012). The ABCB4 gene was mutative in the *Mdr2* ^*−/−*^ mouse, which prevents the mice from secreting phospholipids into the bile. A histological look highly reminiscent of PSC patients results from the retained bile becoming corrosive and destroying cholangiocytes (Smit et al., 1993). The anti-fibrotic effect of GPs on these two models supplied more evidence that GPs ameliorated liver fibrosis induced by different pathogenic processes.

DR is a common typical response to injury in human liver diseases (Sato et al., 2019). Histologically, DR is described as the proliferation of ductular reactive cells, arising from the proliferation of pre-existing cholangiocytes, the differentiation of HPCs, or the biliary metaplasia of hepatocytes, which can exhibit a ductular phenotype and be identified of cholangiocyte markers, CK19 and CK7 (Sato et al., 2019). It is also described as a dynamic and complex process involving many other cells and reactions, such as inflammatory cells, extracellular matrix, and endothelial cells in the reactive lesions (Sato et al., 2019; Coll et al., 2022), which facilitates liver fibrosis and correlates with disease severity (Aguilar-Bravo et al., 2019). In contrast previous studies proposed that the anti-fibrotic effect of GPs may be related to altering glycolysis metabolism and protecting against the damage of aldehydes and lipid peroxidation (Song et al., 2017), and blocking the proliferation of HSCs (Chen et al., 2008), our study found that both in CCl_4_/2-AAF-treated rats and *Mdr2* ^*−/−*^ mice, GPs down-regulated the Epcam, CK19 and CK7 expression, and decreased the number of CK19^+^/OV6^+^, Epcam^+^/CK7^+^ cells, which suggested that GPs suppressed DR. Further studies proved that GPs inhibited the differentiation of WB-F344 cells into a biliary phenotype inducing by SB *in vitro*. Both *in vivo* and *in vitro* results demonstrated that DR, the key pathological factor in liver fibrosis, could be obviously inhibited by GPs.

Hh signaling is vital for hepatic pathophysiology, which consists of four fundamental components: the ligand Hh (containing Shh, Dhh, and Ihh), the receptor Ptch, the signal transducer Smo, and the transcription factor Glis (including Gli1, Gli2, and Gli3). In normal adult livers, Hh signaling is quiescent. Once activated when the liver was injured, Hh signaling can regulate liver sinusoidal endothelial cells capillarization (Wu et al., 2021), stimulate the activation and proliferation of HSCs (Du et al., 2018), and promote biliary expansion (Jalan-Sakrikar et al., 2016), which leads to liver fibrosis (Chung et al., 2016). Thus, inhibiting Hh signaling will be an effective therapeutic target. It has been previously reported liver fibrosis was attenuated by Hh inhibitors, such as cyclopamine (Zhang et al., 2020), vismodegib (Pratap et al., 2012), and GANT61 (Jiayuan et al., 2020). The present study showed that GPs suppressed the activation of Hh signaling both in CCl_4_/2-AAF-treated rats and *Mdr2* ^*−/−*^ mice. Of note, Gli1^+^/CK19^+^ cells were decreased after GPs treatment. Gli1 is one of the strongest downstream transcriptional activators of Hh signaling. Gli1^+^ mesenchymal stem cell-like cells were proved to have a key ability to transition into myofibroblasts (Weiskirchen et al., 2019). Genetic ablation of Gli1^+^ cells substantially improves injury-induced organ fibrosis, which provides a relevant therapeutic target in fibrotic diseases (Kramann et al., 2015). Based on our previous finding, Gli1-dependent Hh signaling triggered the differentiation of HPCs into cholangiocytes and promoted DR-induced fibrosis progression (Chen et al., 2020). The results *in vitro* showed that GPs suppressed the differentiation of WB-F344 cells into a biliary phenotype *via* inhibition of Hh signaling, which was further confirmed in cells transfected with over-expressing Gli1 lentiviral vectors. This is the first time to reveal that GPs alleviate DR and liver fibrosis *via* inhibition of Hh signaling.

NPLC0393 is a natural triterpene saponin constituent of the Chinese herb *Gynostemma pentaphyllum.* NPLC0393 accounts for 2.6% of GPs and is one of the main active ingredients. Previous studies demonstrated that NPLC0393, as a small molecular activator of PP2Cα, successfully ameliorated CCl_4_- and BDL-induced fibrotic models (Wang et al., 2010). And it inhibited the activation of HSCs by regulating TGFβ1/NDRG2/MAPK signaling axis in CCl_4_-induced liver fibrosis (Huang et al., 2020) Our study newly found that NLPC0393 inhibited the differentiation of WB-F344 cells into cholangiocytes in a Gli1-dependent manner. Additional *in vivo* experiments, of course, should be needed to verify the anti-fibrosis and anti-DR of NPLC0393. But the *in vitro* results of the previous study could provide stronger evidence for the important effects of GPs on ameliorating DR and liver fibrosis *via* inhibition of Hh signaling.

## 5 Conclusion

In summary, the present study provided more evidence supporting the therapeutic effect of GPs on liver fibrosis. And for the first time, we demonstrated that GPs alleviated DR and liver fibrosis *via* inhibition of hedgehog signaling. These results will provide new insights into the anti-fibrotic effects of GPs.

## Data Availability

The original contributions presented in the study are included in the article/Supplementary Material, further inquiries can be directed to the corresponding authors.
