# Supplementary figures and images for: Gypenosides ameliorate ductular reaction and liver fibrosis via inhibition of hedgehog signaling
Source: Front Pharmacol. 2022 Nov 22;13:1033103. doi: 10.3389/fphar.2022.1033103 (PMC9722742; doi:10.3389/fphar.2022.1033103)

## Slide 1
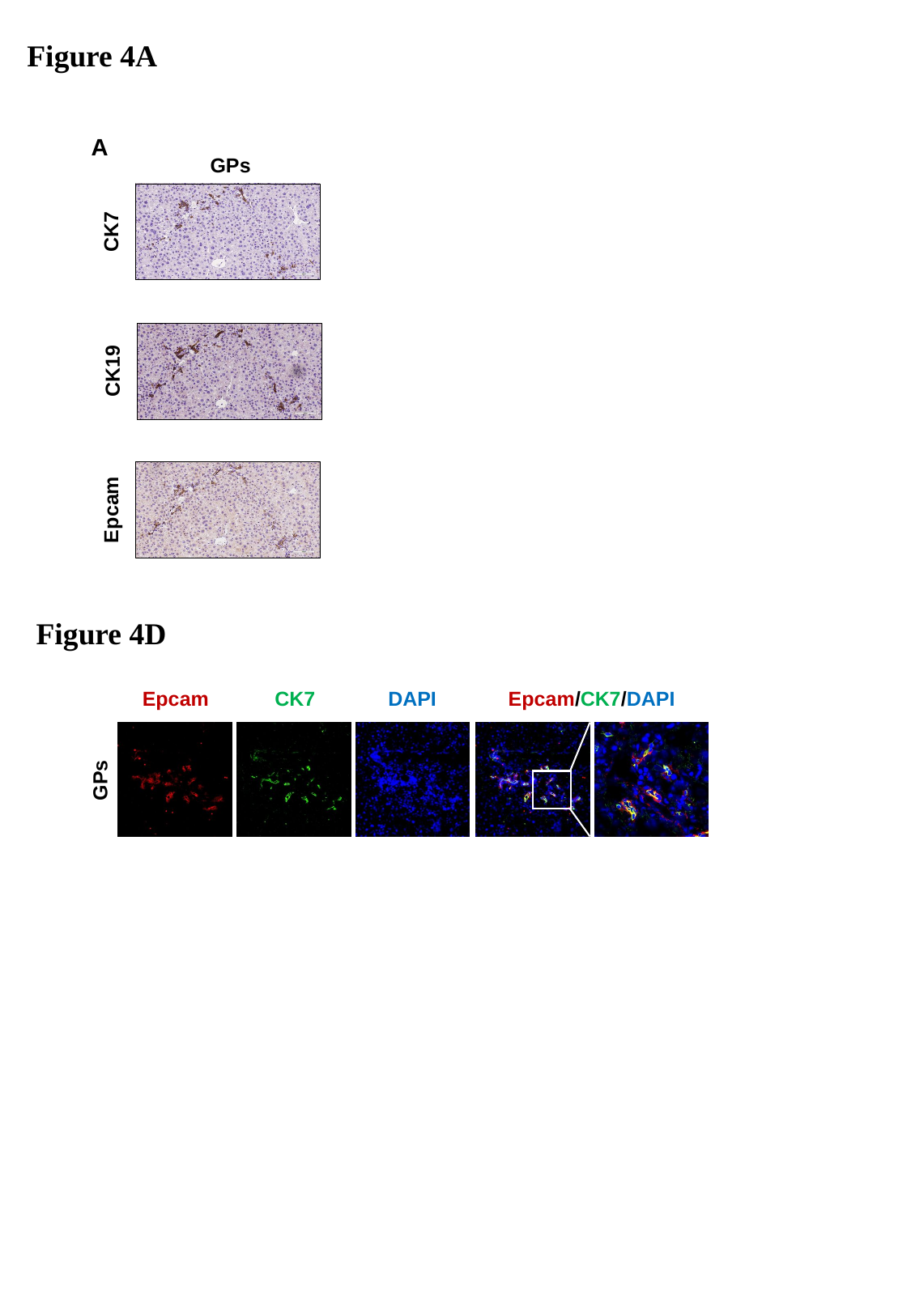

Figure 4A
A
 GPs
CK7
CK19
Epcam
Figure 4D
Epcam
CK7
DAPI
Epcam/CK7/DAPI
 GPs

Supplement: Supplementary file 1 [file Presentation9.PPTX]

## Slide 1
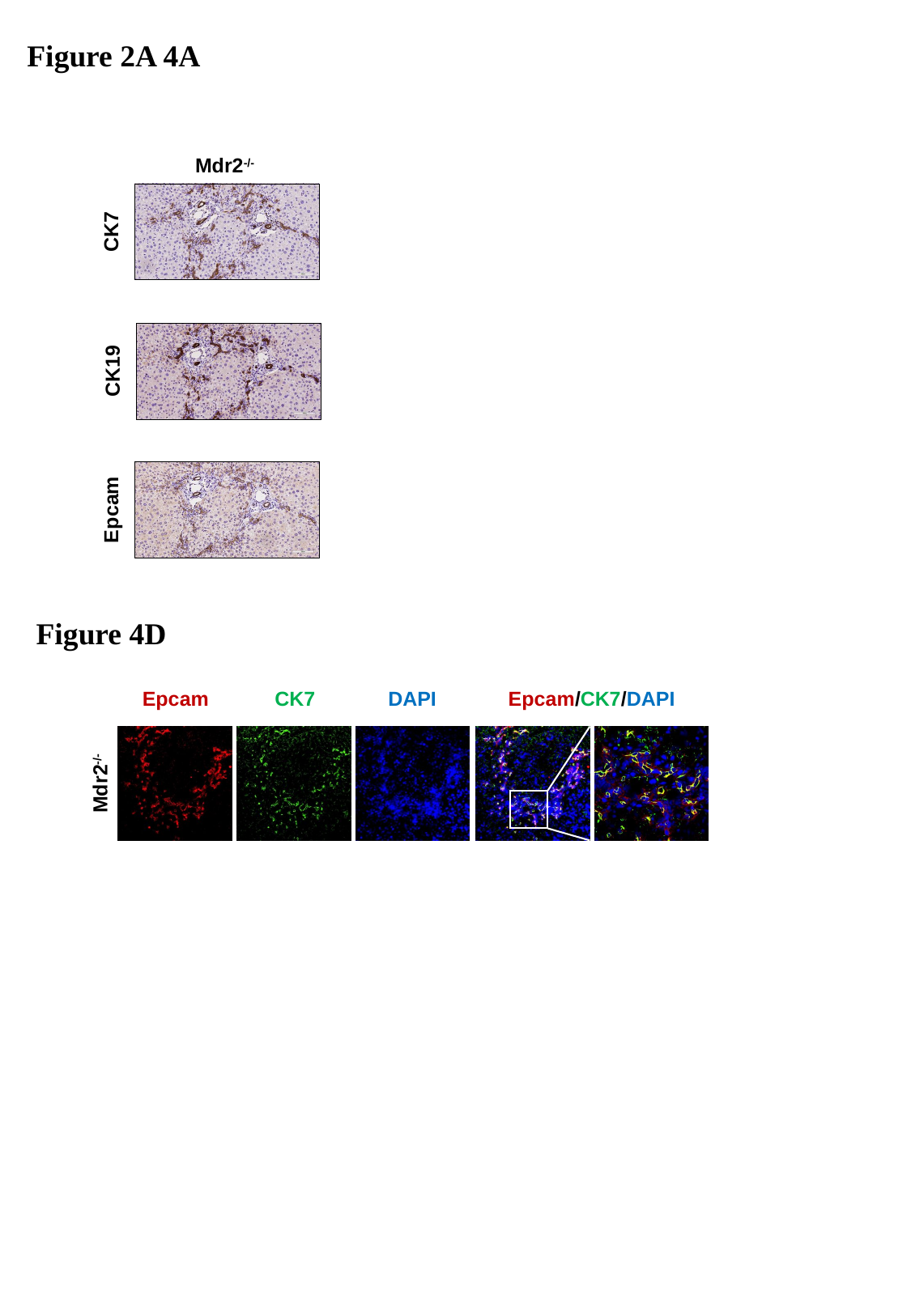

Figure 2A 4A
Mdr2-/-
CK7
CK19
Epcam
Figure 4D
Epcam
CK7
DAPI
Epcam/CK7/DAPI
Mdr2-/-

Supplement: Supplementary file 2 [file Presentation10.PPTX]

## Slide 1
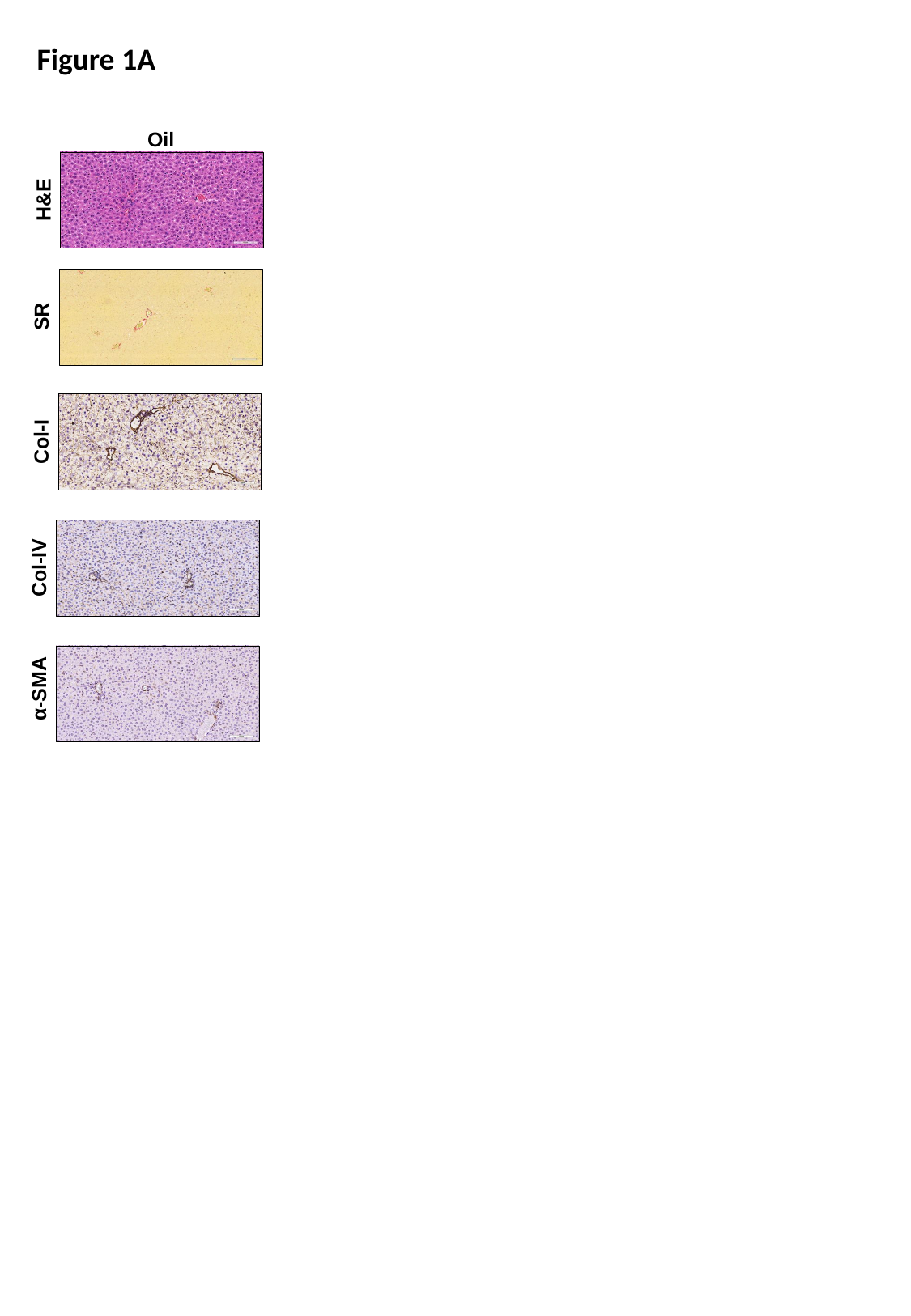

Figure 1A
Oil
H&E
SR
Col-I
Col-IV
α-SMA

Supplement: Supplementary file 3 [file Presentation1.PPTX]

## Slide 1
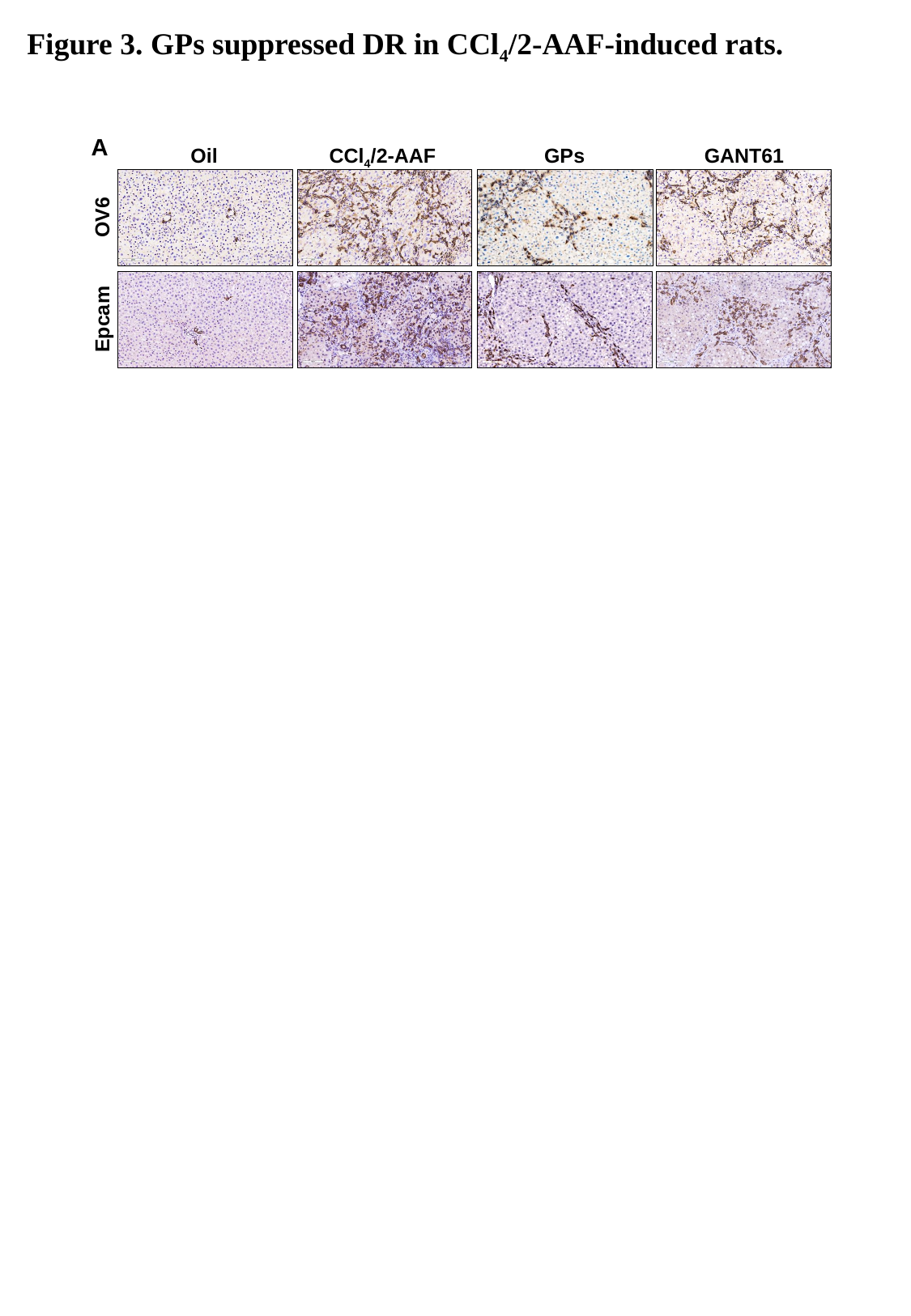

Figure 3. GPs suppressed DR in CCl4/2-AAF-induced rats.
A
Oil
CCl4/2-AAF
GPs
GANT61
OV6
Epcam

Supplement: Supplementary file 4 [file Presentation13.PPTX]

## Slide 1
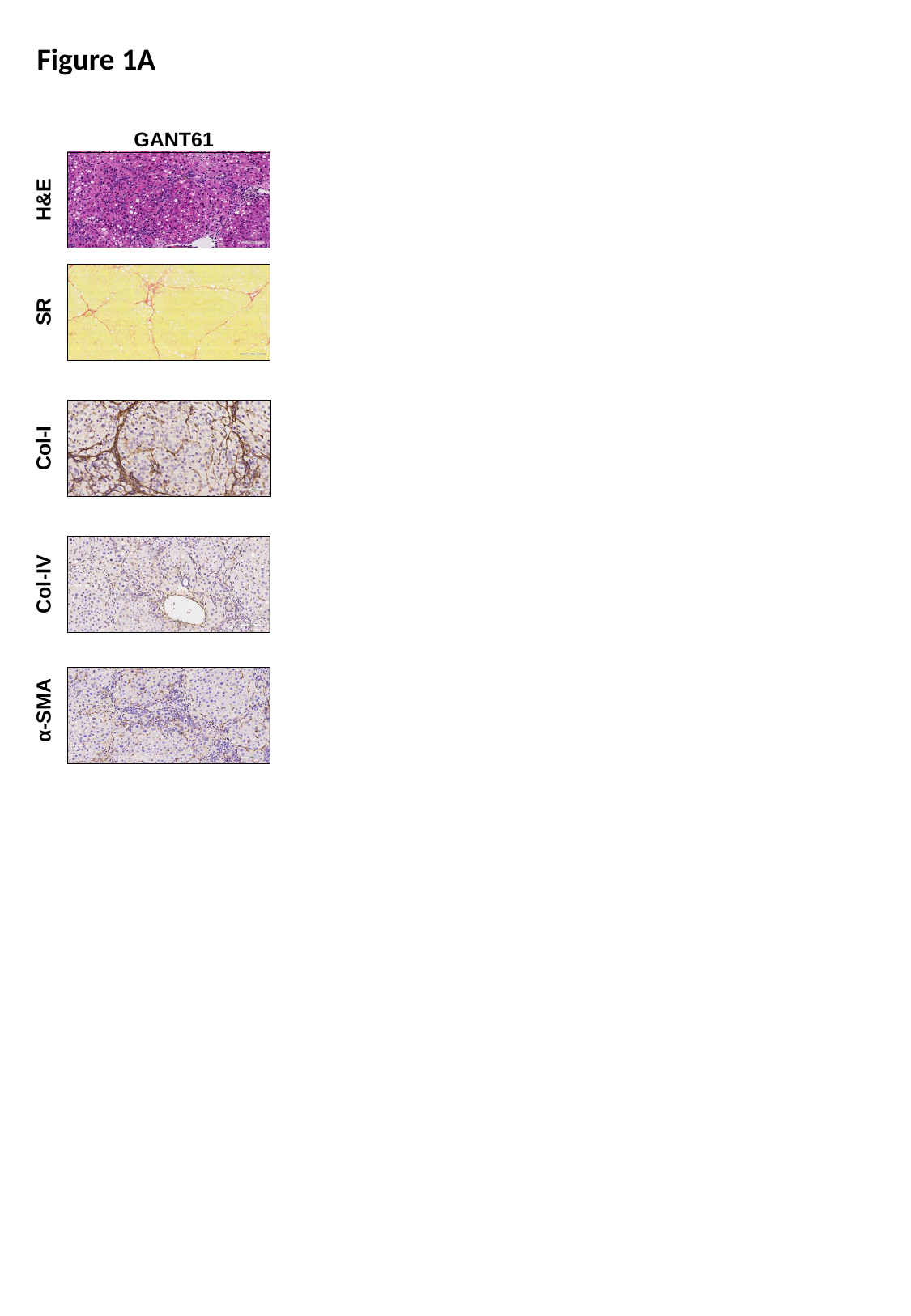

Figure 1A
 GANT61
H&E
SR
Col-I
Col-IV
α-SMA

Supplement: Supplementary file 5 [file Presentation4.PPTX]

## Slide 1
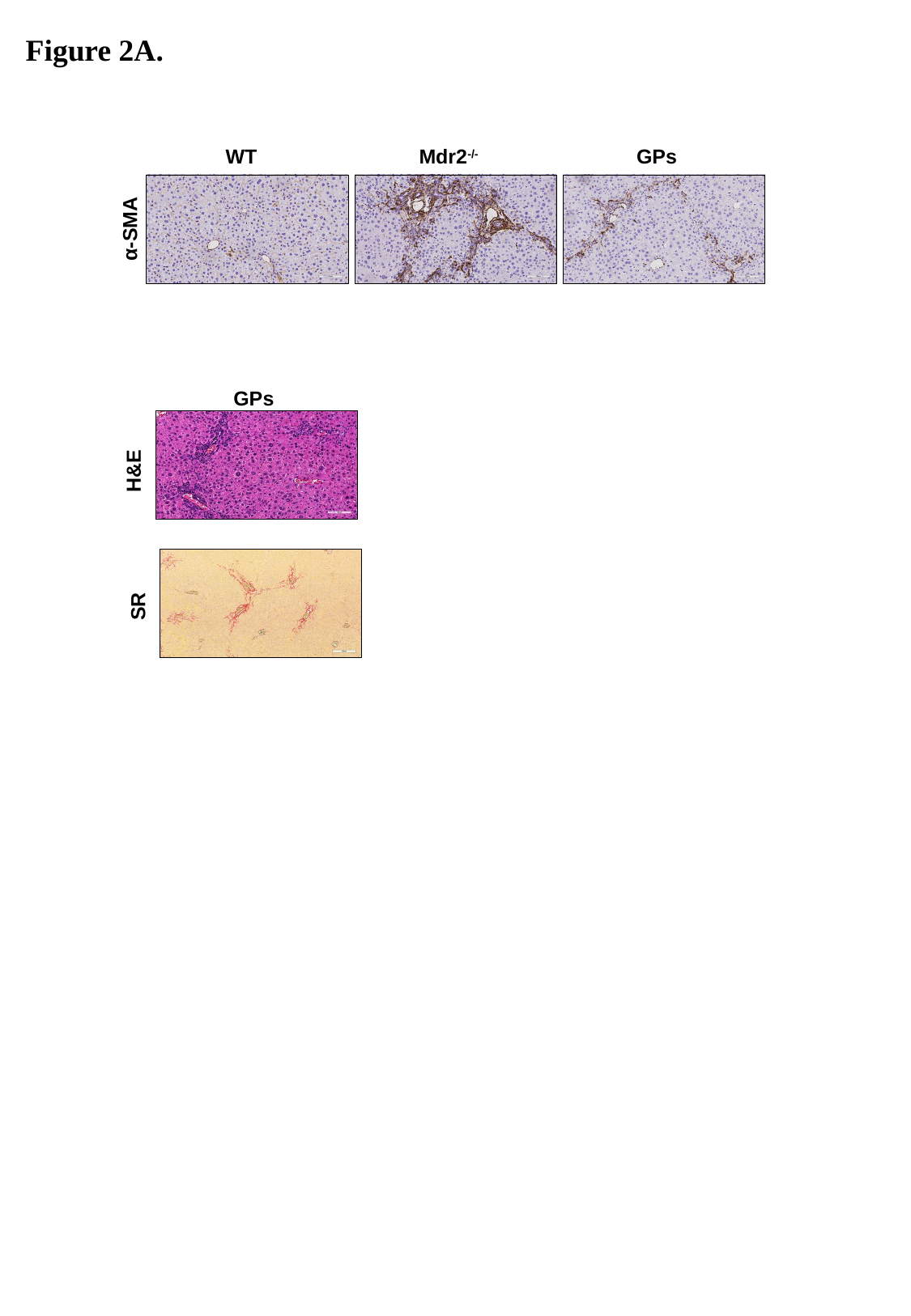

Figure 2A.
WT
Mdr2-/-
GPs
α-SMA
GPs
H&E
SR

Supplement: Supplementary file 6 [file Presentation6.PPTX]

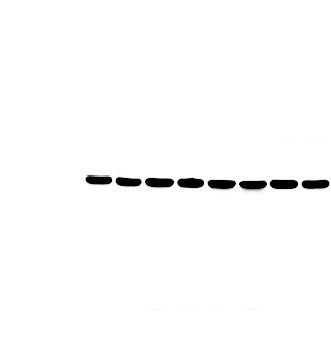

Supplement: Supplementary file 7 [file DataSheet2.ZIP › The original image file for the blots and raw data/Fig.1E-GAPDH.jpg]

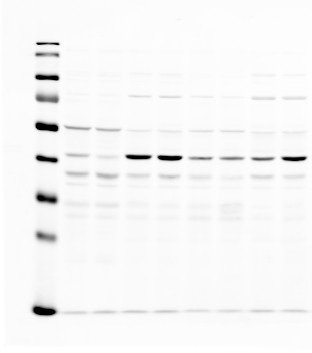

Supplement: Supplementary file 7 [file DataSheet2.ZIP › The original image file for the blots and raw data/Fig.1E-a┴SMA.jpg]

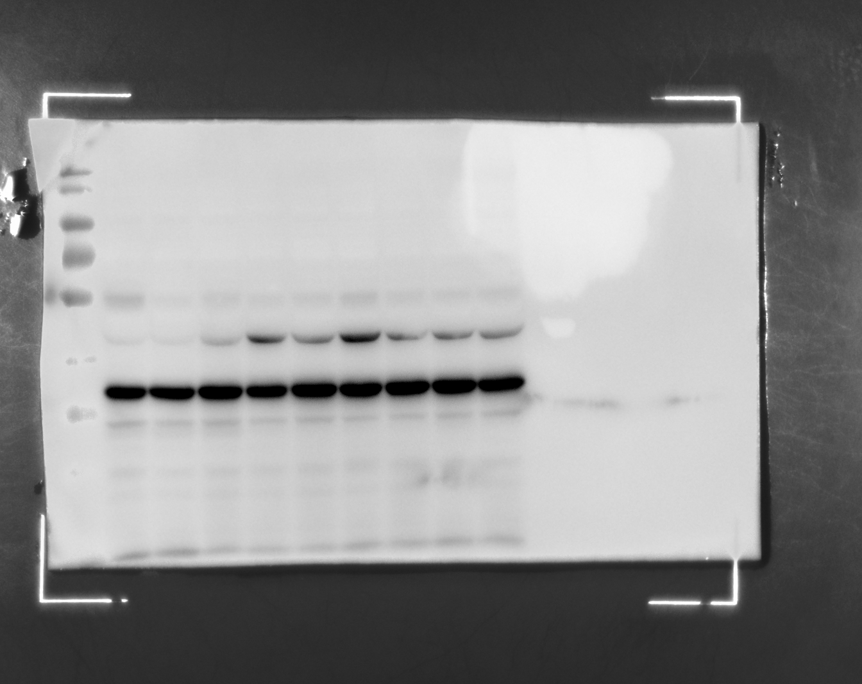

Supplement: Supplementary file 7 [file DataSheet2.ZIP › The original image file for the blots and raw data/Fig.2E-GAPDH.tif]

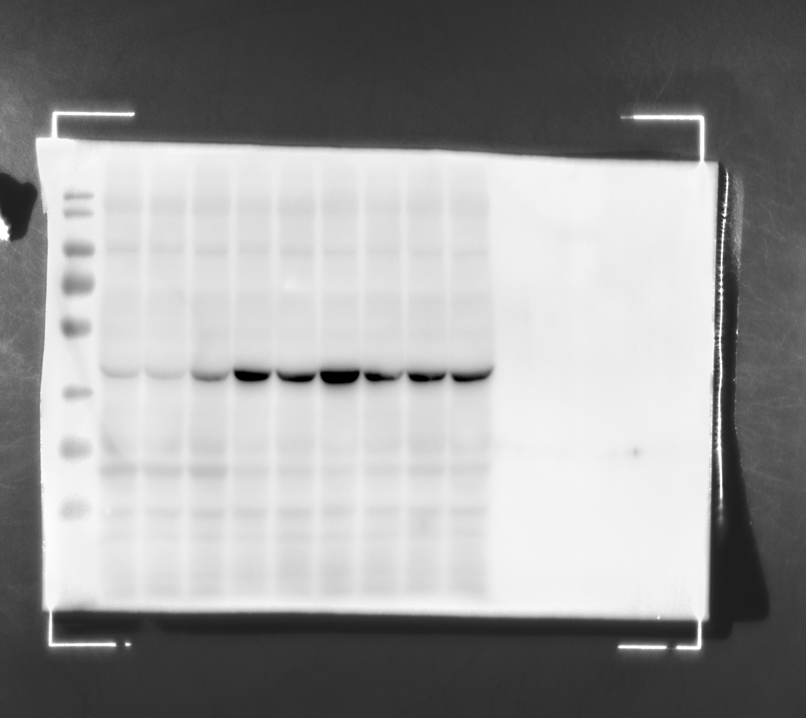

Supplement: Supplementary file 7 [file DataSheet2.ZIP › The original image file for the blots and raw data/Fig.2E-a┴SMA.tif]

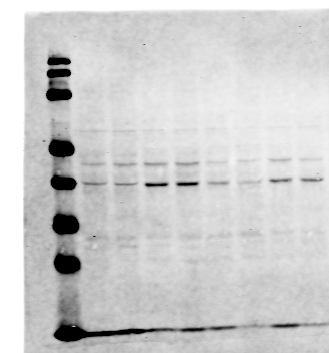

Supplement: Supplementary file 7 [file DataSheet2.ZIP › The original image file for the blots and raw data/Fig.3C-CK19.jpg]

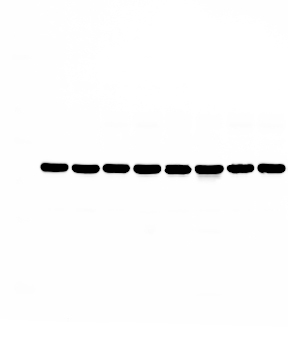

Supplement: Supplementary file 7 [file DataSheet2.ZIP › The original image file for the blots and raw data/Fig.3C-GAPDH.jpg]

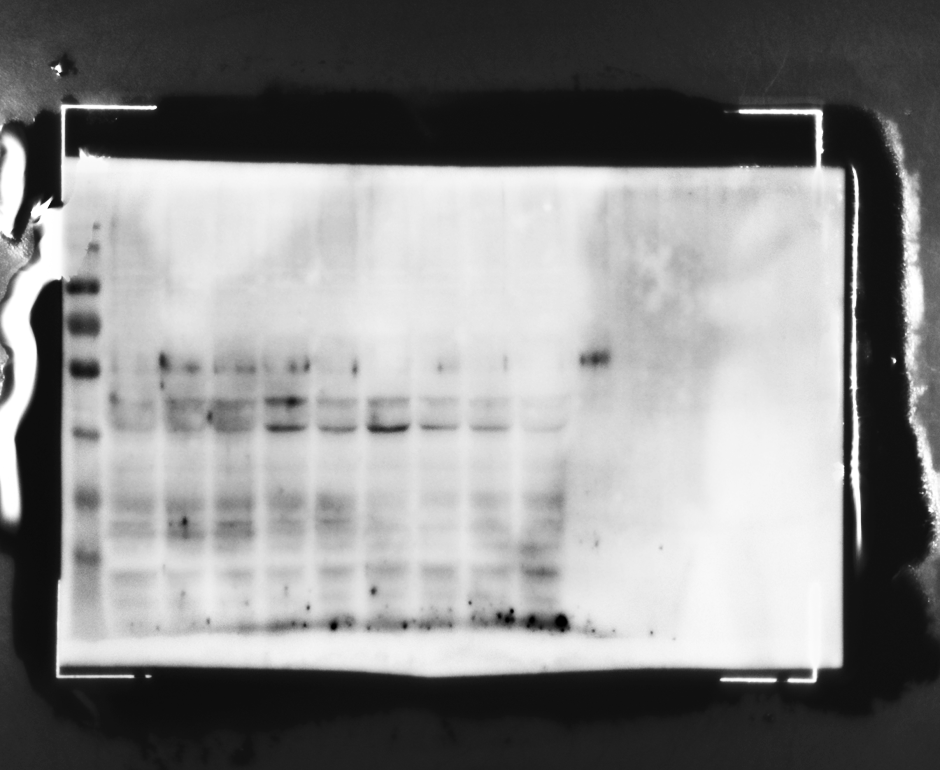

Supplement: Supplementary file 7 [file DataSheet2.ZIP › The original image file for the blots and raw data/Fig.4C-CK19.tif]

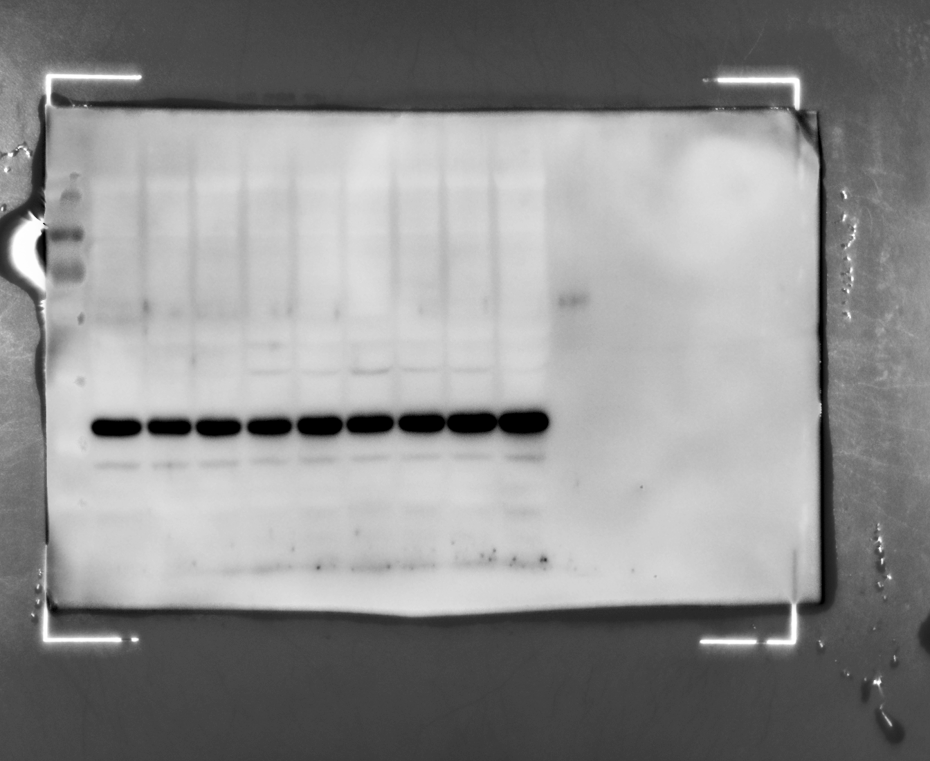

Supplement: Supplementary file 7 [file DataSheet2.ZIP › The original image file for the blots and raw data/Fig.4C-GAPDH.tif]

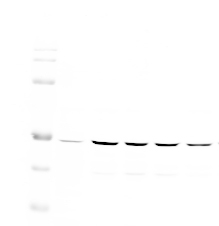

Supplement: Supplementary file 7 [file DataSheet2.ZIP › The original image file for the blots and raw data/Fig.6C-CK19jpg.jpg]

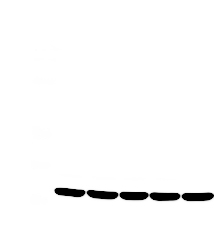

Supplement: Supplementary file 7 [file DataSheet2.ZIP › The original image file for the blots and raw data/Fig.6C-GAPDH.jpg]

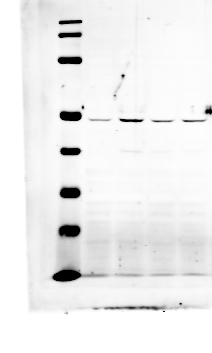

Supplement: Supplementary file 7 [file DataSheet2.ZIP › The original image file for the blots and raw data/Fig.6F-CK19.jpg]

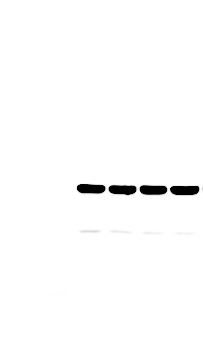

Supplement: Supplementary file 7 [file DataSheet2.ZIP › The original image file for the blots and raw data/Fig.6F-GAPDH.jpg]

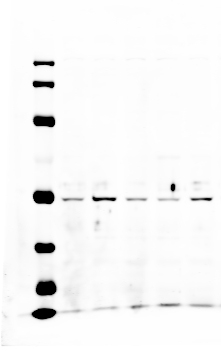

Supplement: Supplementary file 7 [file DataSheet2.ZIP › The original image file for the blots and raw data/Fig.7G-CK19.jpg]

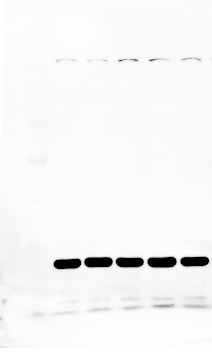

Supplement: Supplementary file 7 [file DataSheet2.ZIP › The original image file for the blots and raw data/Fig.7G-GAPDH.jpg]

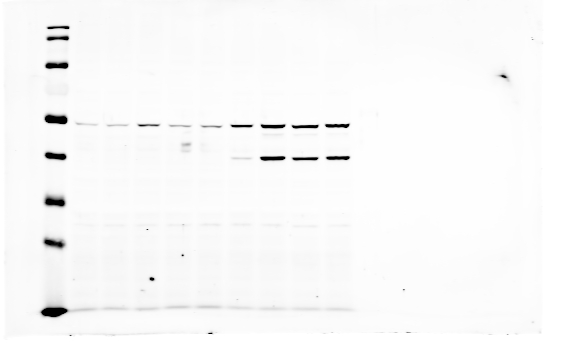

Supplement: Supplementary file 7 [file DataSheet2.ZIP › The original image file for the blots and raw data/Fig.7H-CK19.jpg]

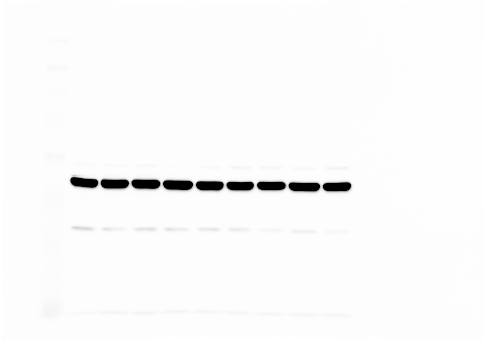

Supplement: Supplementary file 7 [file DataSheet2.ZIP › The original image file for the blots and raw data/Fig.7H-GAPDH.jpg]

## Slide 1
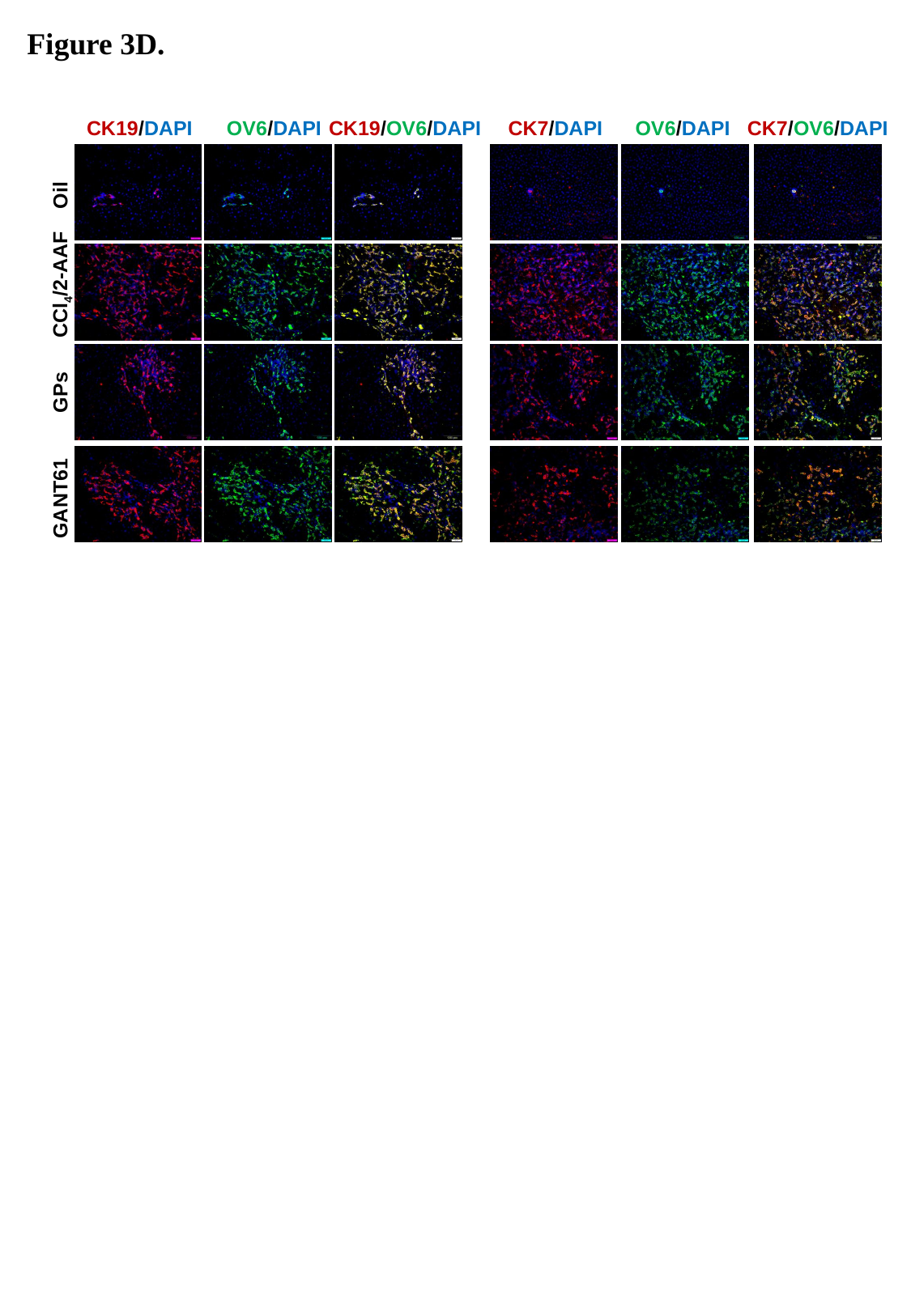

Figure 3D.
CK19/OV6/DAPI
CK7/DAPI
OV6/DAPI
CK7/OV6/DAPI
CK19/DAPI
OV6/DAPI
Oil
CCl4/2-AAF
GPs
GANT61

Supplement: Supplementary file 8 [file Presentation8.PPTX]

## Slide 1
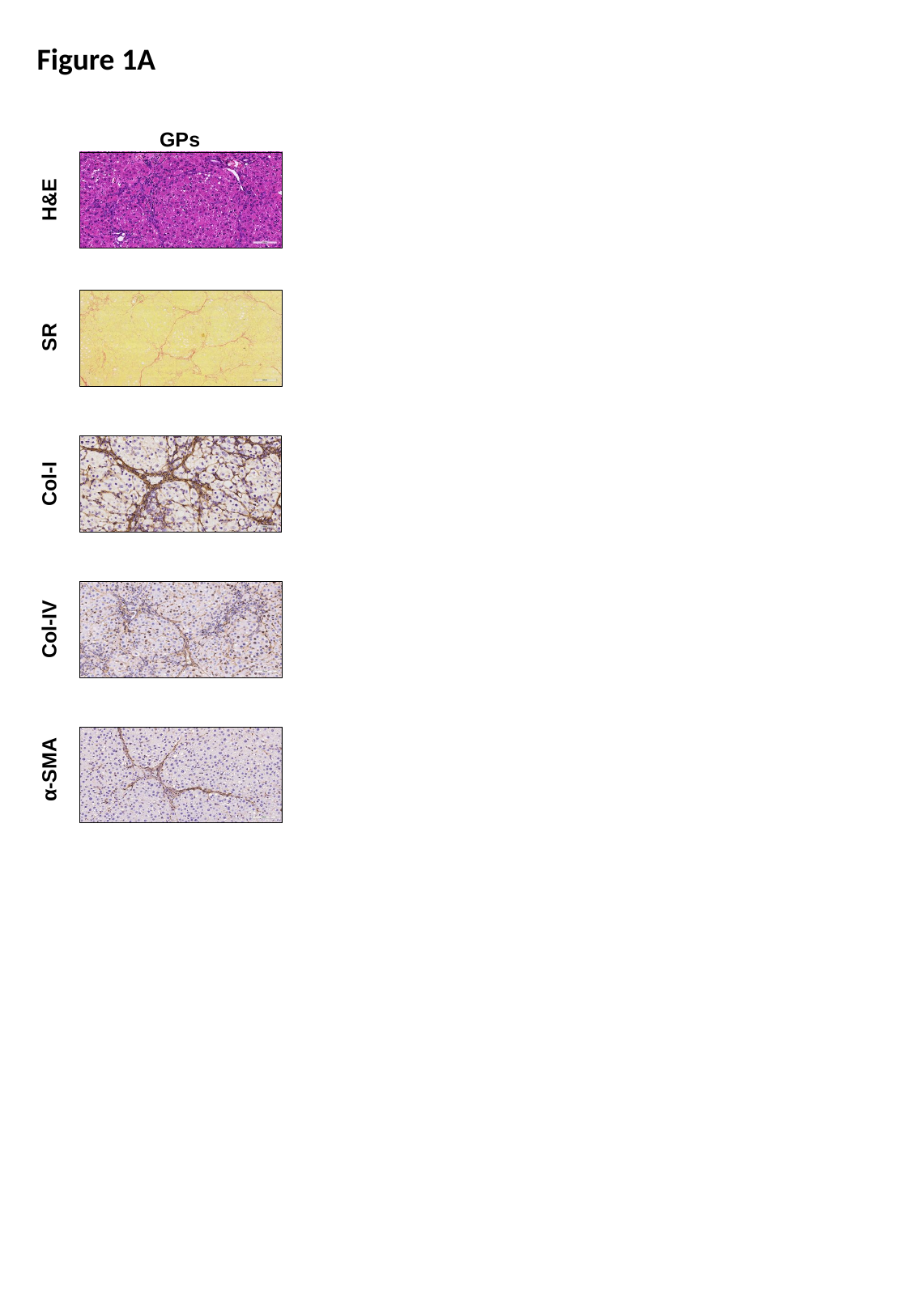

Figure 1A
GPs
H&E
SR
Col-I
Col-IV
α-SMA

Supplement: Supplementary file 9 [file Presentation3.PPTX]

## Slide 1
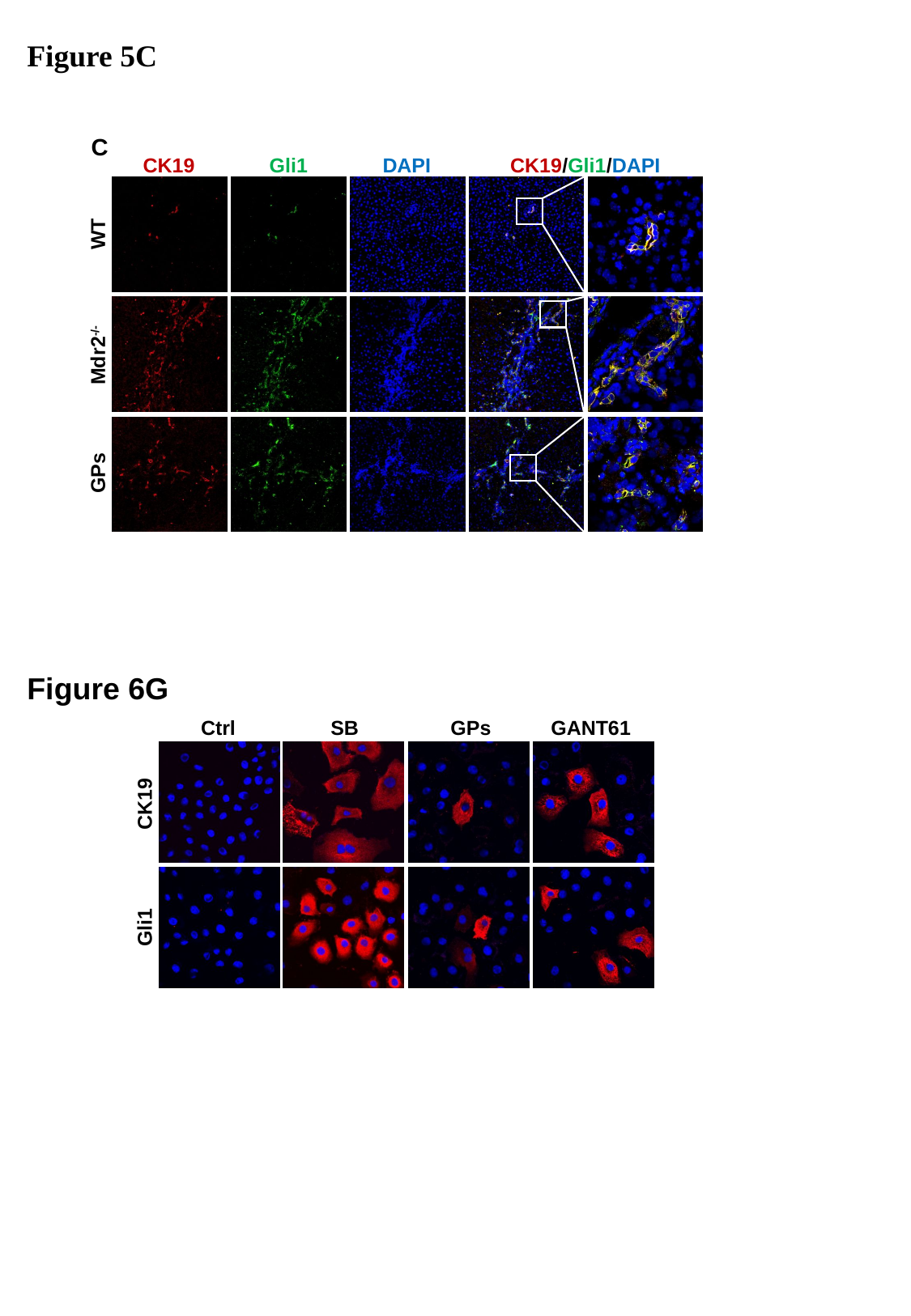

Figure 5C
C
CK19/Gli1/DAPI
CK19
Gli1
DAPI
WT
Mdr2-/-
 GPs
Figure 6G
Ctrl
SB
GPs
GANT61
CK19
Gli1

Supplement: Supplementary file 10 [file Presentation12.PPTX]

## Slide 1
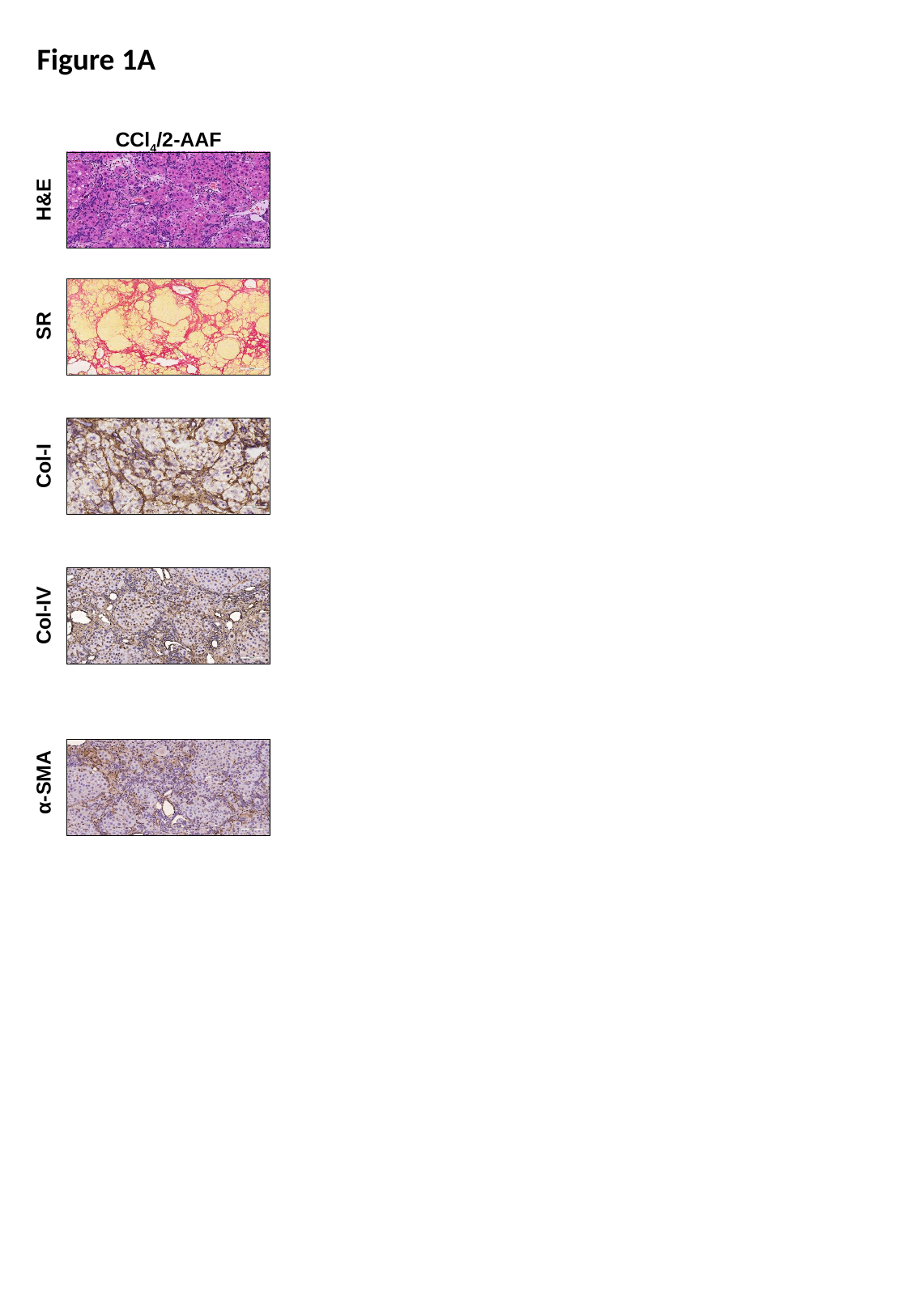

Figure 1A
CCl4/2-AAF
H&E
SR
Col-I
Col-IV
α-SMA

Supplement: Supplementary file 11 [file Presentation2.PPTX]

## Slide 1
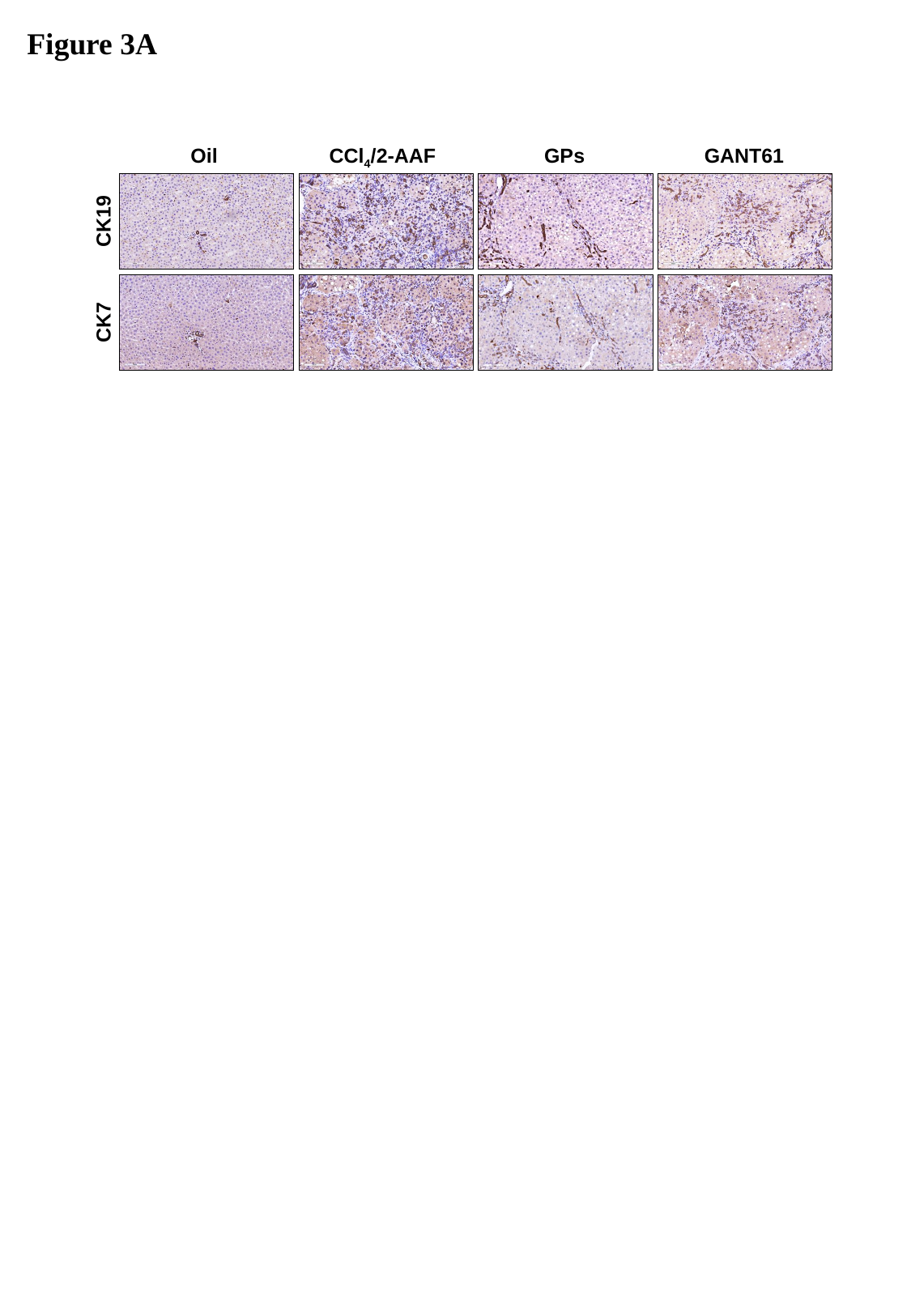

Figure 3A
Oil
CCl4/2-AAF
GPs
GANT61
CK19
CK7

Supplement: Supplementary file 12 [file Presentation7.PPTX]

## Slide 1
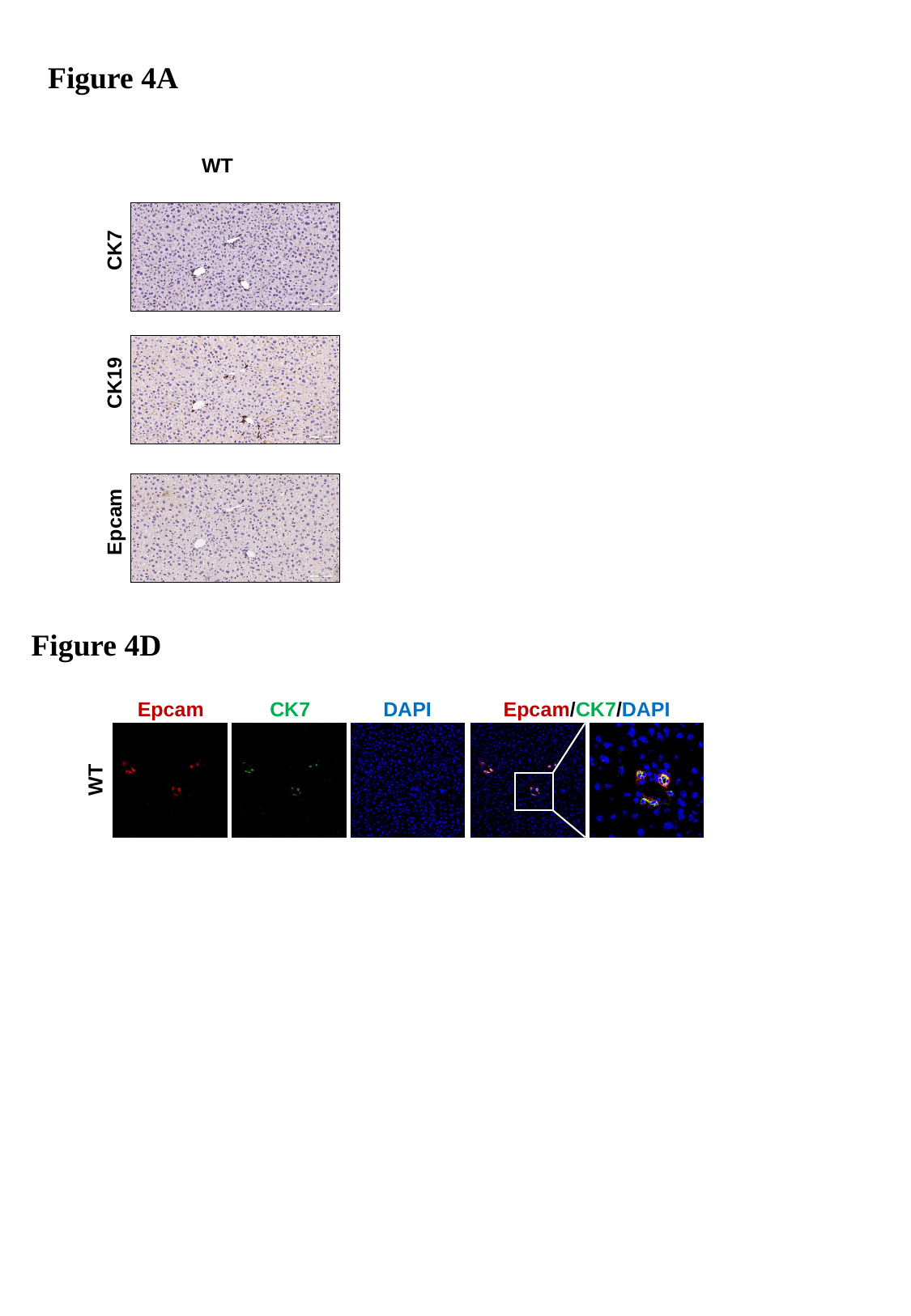

Figure 4A
WT
CK7
CK19
Epcam
Figure 4D
Epcam
CK7
DAPI
Epcam/CK7/DAPI
WT

Supplement: Supplementary file 13 [file Presentation11.PPTX]

## Slide 1
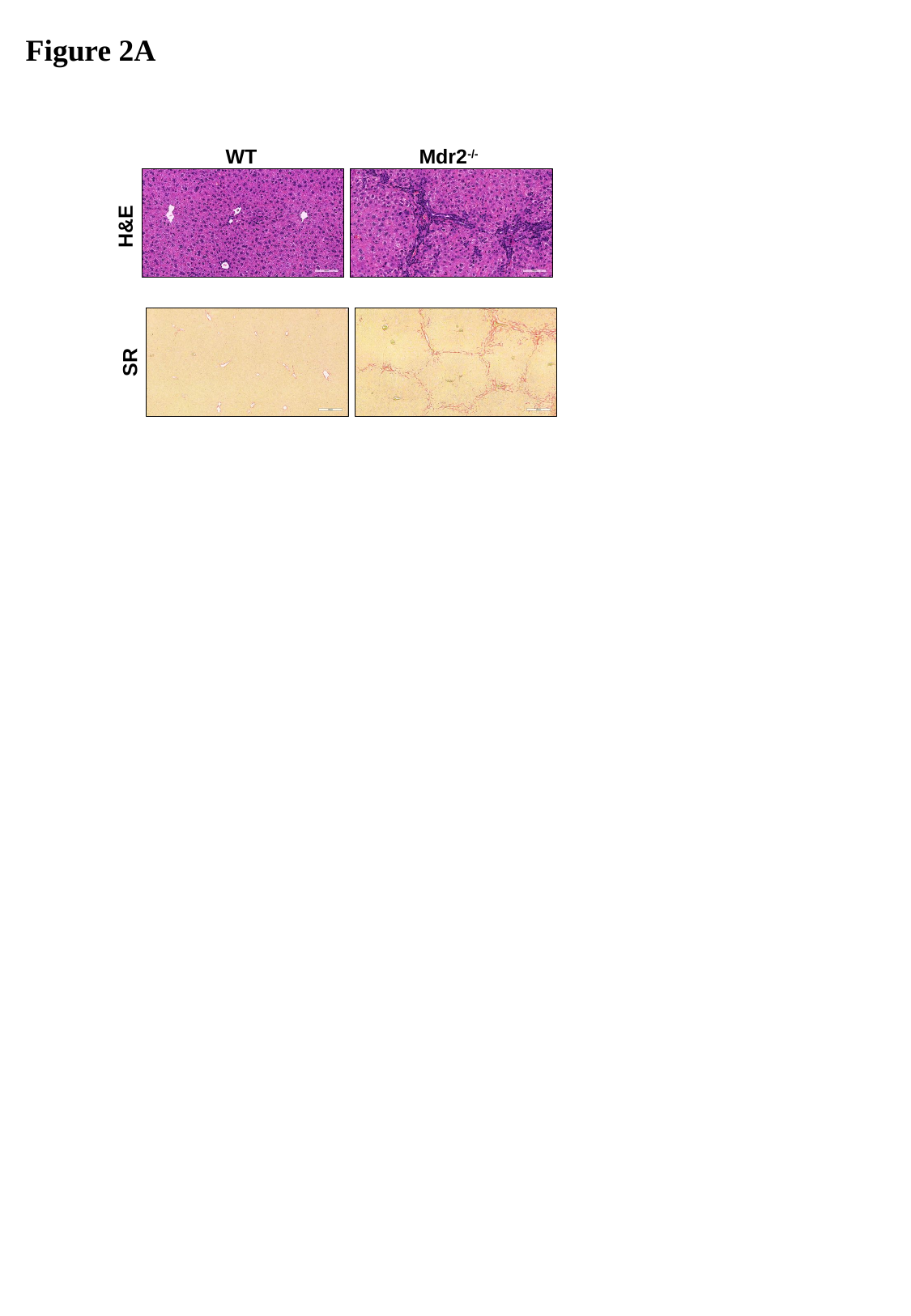

Figure 2A
WT
Mdr2-/-
H&E
SR

Supplement: Supplementary file 15 [file Presentation5.PPTX]
