# Supplementary material for: Gypenosides ameliorate ductular reaction and liver fibrosis via inhibition of hedgehog signaling
Source: Front Pharmacol. 2022 Nov 22;13:1033103. doi: 10.3389/fphar.2022.1033103 (PMC9722742; doi:10.3389/fphar.2022.1033103)
Supplement: Supplementary file 14 [file DataSheet1.docx]

**Supplementary Information**

Gypenosides ameliorate ductular reaction and liver fibrosis via inhibition of hedgehog signaling

**Yonghong Hu^1,2^**†**, Xiaoli He^1,2^**†**, Xiaoxi Zhou^1,2^, Yue Liang^1,2^, Yadong Fu^3^, Linzhang Zhang^3^, Jing Fang^1,2^, Wei Liu^1,2^, Gaofeng Chen^1,2^, Yongping Mu^1,2^, Hua Zhang^1,2^, Chenghai Liu^1,2^, Ping Liu^1,2,3^*, Jiamei Chen^1,2^***

1. Institute of Liver diseases, Key Laboratory of Liver and Kidney Diseases (Ministry of Education), Shuguang Hospital affiliated to Shanghai University of Traditional Chinese Medicine, Shanghai, China

2. Shanghai Key Laboratory of Traditional Chinese Clinical Medicine, Shanghai, China

3. Institute of Interdisciplinary Integrative Medicine Research, Shanghai University of Traditional Chinese Medicine, Shanghai, China

† These authors contributed equally to this work and share first authorship.

*** Corresponding Author:** Jiamei Chen, [cjm0102@126.com](mailto:cjm0102@126.com); Ping Liu, [liuliver@vip.sina.com](mailto:liuliver@vip.sina.com).

**Supplementary Table 1: Antibodies for Immunohistochemical staining**

| Antibodies | Manufacturer | Catalog# | Dilution |
| --- | --- | --- | --- |
| Col-Ⅰ | abcam | ab34710 | 1:200 |
| Col-Ⅳ | abcam | ab6586 | 1:200 |
| α-SMA | abcam | ab124964 | 1:1000 |
| OV6 | Santa Cruz Biotechnology | sc-101863 | 1:800 |
| CK19 | Proteintech | 10712-1-AP | 1:400 |
| CK7 | Proteintech | 15539-1-AP | 1:400 |
| Epcam | abcam | ab71916 | 1:800 |

**Supplementary Table 2: Antibodies for Immunofluorescence staining**

| Antibodies | Manufacturer | Catalog# | Dilution |
| --- | --- | --- | --- |
| OV6 | Santa Cruz Biotechnology | sc-101863 | 1:800 |
| CK19 | Proteintech | 10712-1-AP | 1:400 |
| CK7 | Proteintech | 15539-1-AP | 1:400 |
| Epcam | abcam | ab71916 | 1:800 |
| CK7 | Ab9021 | ab9021 | 1:400 |
| Gli1 | R&D | AF3455 | 1:50 |
| Gli1 | Abclonal | A14965 | 1:200 |
| DAPI | Beyotime Biotechnology | C1002 | 1:1000 |
| Goat anti-rabbit Cy3 | abcam | ab6939 | 1:2000 |
| Goat anti-mouse Alexa Fluor 647 | abcam | ab150115 | 1:500 |
| Donkey anti-goat Alexa Fluor 488 | abcam | ab150129 | 1:500 |

**Supplementary Table 3: Antibodies for Western blot**

| Antibodies | Manufacturer | Catalog# | Dilution |
| --- | --- | --- | --- |
| α-SMA | abcam | Ab5694 | 1:500 |
| CK19 | Proteintech | 10712-1-AP | 1:1000 |
| GAPDH | Proteintech | 60004-I-Ig | 1:5000 |
| Anti-Mouse IgG(H+L) (DyLight^TM^ 800 4× peg Conjugante) | Cell signaling Technology | 5257 | 1:10000 |
| Anti-Rabbit IgG(H+L) (DyLight^TM^ 680 Conjugante) | Cell signaling Technology | 5366 | 1:10000 |
| HRP labeled goat anti-mouse IgG(H+L) | Beyotime | A0216 | 1:1000 |
| HRP labeled goat anti-mouse IgG(H+L) | Beyotime | A0208 | 1:1000 |

**Supplementary Table 4: Primer sequences for rat**

| Gene name | Forward | Reverse |
| --- | --- | --- |
| *Acta2* | AGACCTTCAATGTCCCTGCCA | GTTGTGAGTCACGCCATCTCC |
| *Col1a1* | TGTCTGGTTTGGAGAGAGCA | AGTGATAGGTGATGTTCTGG |
| *Col4* | TTTCCAGGGTTACAAGGTGT | AGTCCAGGTTCTCCAGCATC |
| *Epcam* | GGCGTGGAACTCAGAACTTA | TCTACTGTGGGCTGTTTATG |
| *Ck19* | CAGGTCGCTGTCCACACTA | TATCTCTGCCACAGTGCCTT |
| *Ck7* | CGGAATGAGATTGCGGAGAT | CCTTGTTCCTCAGCCTCTGC |
| *Dhh* | GCCCCAATCTGTCAGGAATG | AAGTCCCACCCCACTCAAAG |
| *Shh* | GTCGAGCAGTGGACATCACC | AGAGCAGTGGATGCGAGCTT |
| *Ihh* | CCCTCGTCTTGGTGTAGAG | GAATCGCAGTCAGAGCTAGC |
| *Smo* | TCCAGCGAGACCCTATCCT | AACCACACTACTCCAGCCAT |
| *Ptch1* | TAGTCTGCCGCTGCCAACTT | GCCCTGTGAGGTCTCTGTGA |
| *Ptch2* | TAGTCTGCCGCTGCCAACTT | GCCCTGTGAGGTCTCTGTGA |
| *Gli1* | AGCATCACCGAAAATGTTG | TATCCCAGAGTGTCAGCAGA |
| *Gli2* | GACCTGACCCCCTTGTTCT | CAGCGGACCTGGATTCAT |
| *Gli3* | CAAGGCTTTCTCTAACGCTT | GGGTCTGTGTAACGCTTGGT |

**Supplementary Table 5: Primer sequences for mouse**

| Gene name | Forward | Reverse |
| --- | --- | --- |
| *Acta2* | AATGGCTCTGGGCTCTGTAA | TCTCTTGCTCTGGGCTTCAT |
| *Col1a1* | CCTCAGGGTATTGCTGGACAAC | CAGAAGGACCTTGTTTGCCAGG |
| *Col3* | GACCAAAAGGTGATGCTGGAC | CAAGACCTCGTGCTCCAGTTAG |
| *Col4* | ATGGCTTGCCTGGAGAGATAGG | TGGTTGCCCTTTGAGTCCTGGA |
| *Tgf-β1* | AGTCAGAGACGTGGGGACTT | CGGAATAGGGGCGTCTGAG |
| *Epcam* | GAGTCCGAAGAACCGACAAGGA | GATGTGAACGCCTCTTGAAGCG |
| *Ck19* | ACCTACCTTGCTCGGATTG | TGACTTCGGTCTTGCTTAT |
| *Ck7* | CGGAGATGAACCGCTCTATCCA | CATGAGCATCCTTGATTGCCAGC |
| *Dhh* | GCAGACCGCCTGATGACAGA | GTTCATCACCGCGATGGCTA |
| *Smo* | AAGGCCACCCTGCTCATCTG | AGGCCTTGGCGATCATCTTG |
| *Ptch2* | TGGCAATGATGACTGTGGAG | TACCATGGCTGGTCAGGAA |
| *Gli1* | CTCAAACTGCCCAGCTTAACCC | TGCGGCTGACTGTGTAAGCAGA |
| *Gli2* | ACACTGTGGAGGACTGCCTACA | GGCATCTCCATGCCACTGTCAT |
